# B Cell-Intrinsic Role for IRF5 in TLR9/BCR-Induced Human B Cell Activation, Proliferation, and Plasmablast Differentiation

**DOI:** 10.3389/fimmu.2017.01938

**Published:** 2018-01-10

**Authors:** Saurav De, Baohong Zhang, Tiffany Shih, Sukhwinder Singh, Aaron Winkler, Robert Donnelly, Betsy J. Barnes

**Affiliations:** ^1^Rutgers Graduate School of Biomedical Sciences, Newark, NJ, United States; ^2^Center for Autoimmune Musculoskeletal and Hematopoietic Diseases, The Feinstein Institute for Medical Research, Manhasset, NY, United States; ^3^Clinical Genetics and Bioinformatics, Pfizer Inc., Cambridge, MA, United States; ^4^Department of Pathology and Laboratory Medicine, Rutgers Biomedical and Health Sciences, New Jersey Medical School, Newark, NJ, United States; ^5^Department of Inflammation and Immunology, Pfizer Inc., Cambridge, MA, United States; ^6^Rutgers Biomedical and Health Sciences, New Jersey Medical School–Cancer Center, Newark, NJ, United States

**Keywords:** interferon regulatory factor 5, human primary B cells, plasmablasts, autoantibodies, immunoglobulin G, differentiation, toll-like receptor

## Abstract

Upon recognition of antigen, B cells undergo rapid proliferation followed by differentiation to specialized antibody secreting cells (ASCs). During this transition, B cells are reliant upon a multilayer transcription factor network to achieve a dramatic remodeling of the B cell transcriptional landscape. Increased levels of ASCs are often seen in autoimmune diseases and it is believed that altered expression of regulatory transcription factors play a role in this imbalance. The transcription factor interferon regulatory factor 5 (IRF5) is one such candidate as polymorphisms in *IRF5* associate with risk of numerous autoimmune diseases and correlate with elevated *IRF5* expression. *IRF5* genetic risk has been widely replicated in systemic lupus erythematosus (SLE), and loss of *Irf5* ameliorates disease in murine lupus models, in part, through the lack of pathogenic autoantibody secretion. It remains unclear, however, whether IRF5 is contributing to autoantibody production through a B cell-intrinsic function. To date, IRF5 function in healthy human B cells has not been characterized. Using human primary naive B cells, we define a critical intrinsic role for IRF5 in B cell activation, proliferation, and plasmablast differentiation. Targeted IRF5 knockdown resulted in significant immunoglobulin (Ig) D retention, reduced proliferation, plasmablast differentiation, and IgG secretion. The observed decreases were due to impaired B cell activation and clonal expansion. Distinct from murine studies, we identify and confirm new IRF5 target genes, *IRF4, ERK1*, and *MYC*, and pathways that mediate IRF5 B cell-intrinsic function. Together, these results identify IRF5 as an early regulator of human B cell activation and provide the first dataset in human primary B cells to map IRF5 dysfunction in SLE.

## Introduction

Antibody secreting cells (ASCs), referred to as plasmablasts and plasma cells, are critical mediators of adaptive immunity and are often found elevated in the circulation of patients with systemic lupus erythematosus (SLE) (1–4). ASCs release high titers of antibody capable of neutralizing invading antigen and stem from naive and memory B cells that have been activated through antigen recognition. Differentiation of naive B cells to ASCs requires multiple B cell activation pathways, including B cell receptor (BCR), T cell-mediated cytokine signaling, and toll-like receptors (TLRs) (5, 6). BCR activation occurs through binding of cognate antigen, leading to activation of distal signaling cascades, including the Ras-Raf-MEK-ERK1/2 kinase cascade and the Btk-PI3K-PLC-NFĸB cascade, ultimately culminating in transcriptional activation of proliferation and prosurvival genes (7, 8). Complimentary T cell activation allows for interaction between B and T cells. Engagement of the respective B and T cell surface proteins, CD40 and CD40 ligand (CD40L), further enhances transcription of B cell survival and proliferation genes. T cell-mediated cytokine signaling acts to drive ASC differentiation, as well as to specify the antibody subclass secreted. TLR signaling can act synergistically with both T cell-dependent and -independent B cell activation pathways and represents a unique bridge between innate and adaptive immune responses (9).

These various B cell activation pathways utilize a network of transcription factors to drive proliferation, survival and differentiation (3). Immediately following activation, B cells rapidly proliferate to ensure sufficient numbers of antigen-specific B cells to undergo ASC differentiation. This initial burst of proliferation is dependent on the transcription factor *MYC* (10, 11). Following several rounds of proliferation, B cells upregulate interferon regulatory factor 4 (IRF4), BLIMP1, and XBP1, which control ASC differentiation and prepare the cell for antibody secretion (3, 12–14). In response to IRF4 upregulation, B cells undergo terminal chromosomal rearrangement of the immunoglobulin (*Ig*) locus known as class switch recombination (CSR) (12). CSR results in the switching of antibody subtype from IgM to IgG, IgA, or IgE, and is dependent on the enzyme activation-induced deaminase (AID). The majority of work on ASC differentiation has occurred in mice as human primary B cells are notoriously difficult to manipulate (by knockdown or overexpression) for functional analyses.

Genetic variants in or near the transcription factor interferon regulatory factor 5 (*IRF5*) have been robustly associated with SLE risk and elevated IRF5 expression and activation have been reported in SLE immune cells (15–19). Mice lacking *Irf5* are protected from murine lupus disease onset and severity (20–23). A common finding between the different models of murine lupus that lack *Irf5* is the significant decrease in pathogenic autoantibody secretion suggesting a role for IRF5 in B cells. In mice, *Irf5* was found to regulate *IL6, PRDM1*, and *IgG2a* expression (22, 24–26). SLE pathogenesis is associated with polyclonal B cell hyperreactivity resulting in an autoreactive B cell repertoire, elevated circulating ASCs and autoantibodies (2, 27). Whether IRF5 contributes to ASC differentiation or antibody production in human primary B cells is not known.

Here, we developed a method of targeted gene knockdown in human primary naive B cells. While IRF5 expression and activity have been well-characterized in human monocytes and dendritic cells, its role in B cells remains to be defined (19, 28–35). We show that IRF5 is required in the early stages of B cell activation and proliferation in response to TLR9/BCR-induced ASC differentiation. IRF5 knockdown resulted in a significant increase in the number of IgD^+^ B cells, reduced activation, clonal expansion, plasmablast differentiation, and IgG1/3 secretion. Distinct from murine studies, we identify and confirm new IRF5 target genes, *IRF4, ERK1*, and *MYC*, that mediate IRF5 B cell-intrinsic function.

## Materials and Methods

### Naive B Cell Isolation

Blood was drawn *via* peripheral phlebotomy and PBMC isolated by Ficoll centrifugation (18). PBMC were diluted to a concentration of 5 × 10^7^ cells/mL and naive B cells isolated using Stem Cell Technologies Kit (Cat#: 19254). Magnetic separation was performed to achieve a >95% enriched population of naive B cells (CD19^+^CD20^+^IgD^+^CD27^−^), as determined by flow cytometry (Figure S1A in Supplementary Material). This study was carried out in accordance with the recommendations of the Rutgers Biomedical and Health Sciences IRB and the Feinstein Institute for Medical Research IRB with written informed consent from all subjects. All subjects gave written informed consent in accordance with the Declaration of Helsinki. The protocol was approved by the Rutgers Biomedical and Health Sciences IRB and the Feinstein Institute for Medical Research IRB. The Ramos lymphoblastic B cell line was purchased from ATCC^®^ and cultured in RPMI-1640 with 10% fetal bovine serum.

### Imaging Flow Cytometry Analysis of IRF5 Activation

Isolated PBMC were stained for CD19 (BD Biosciences #562847) and fixed overnight in 1% paraformaldehyde. Cells were permeabilized the following day in 0.01% Triton-X-100 and stained for intracellular IRF5 (Abcam #ab193245) (19). Images were acquired on the Amnis ImageStream X Mark II imaging flow cytometer using the 40× objective. Nuclear translocation was quantified in the Amnis IDEAS software suite using the similarity score feature (Figure S1B in Supplementary Material).

### IRF5 siRNA Nucleofection

Isolated naive B cells (3 × 10^6^) were resuspended in Amaxa buffer P3 (Lonza: #V4XP-3032) and distributed to Amaxa 100 µL cuvettes. B cells were nucleofected with 500 nM of mock, ON-TARGETplus non-targeting control pool (GE Dharmacon: #D-001810-10-05), or SMARTpool ON-TARGETplus human IRF5 siRNA (GE Dharmacon: #L-011706-00-0010). Cells were nucleofected on the Amaxa 4D Nucleofector using program EO-117 and then immediately added to 1 mL of RPMI 1640 (+10% FBS, 1× glutamine, 1× non-essential amino acids) and cultured for 24 h, pelleted and re-nucleofected with siRNA. For GFP co-nucleofection, pmaxGFP™ Vector (Lonza) or GFP mRNA (Trinity Biotech: #L6101) was titrated over a concentration range with 500 nM *IRF5* siRNA; 15 µg GFP mRNA gave the best results.

### qRT-PCR and Western Blotting

RNA was isolated with Trizol^®^ and qRT-PCR performed as described (18) with primer sets: *MYC* 5′-CCTGGTGCTCCATGAGGAGAC, 3′-CAGACTCTGACCTTTTGCCAGG; *IRF4* 5′-GAACGAGGAGAAGAGCATCTTCC, 3′-CGATGCCTTCTCGGAACTTTCC; *IL6* 5′-AGACAGCCACTCACCTCTTCAG, 3′-TTCTGCCAGTGCCTCTTTGCTG; *PRDM1* 5′-AGAAGGCTCCAGCCATCTCTGT, 3′-TGCTGGTAGAGTTCGGTGCAGA. Threshold values (*C*_T_) were averaged over each sample replicate, followed by normalization *via* the ΔΔ*C*_T_ method to β-actin. For Western blot analysis, 2 days postnucleofection, naive B cells were stimulated with mock or anti-IgM^+^ CpG-B for 24 h and then harvested for lysate preparation in RIPA buffer (10 nM Tris–HCl pH 8.0, 1 mM EDTA, 1% Triton X-100, 0.1% Sodium Deoxycholate, 0.1% SDS, and 140 mM NaCl) (36).

### *In Vitro* B Cell Activation and Plasmablast Differentiation

Isolated naive B cells were cultured in 96-well U-bottom plates at a minimal density of 1 × 10^6^ with either 150 ng/mL CD40L (Peprotech #310-02) alone or with 100 ng/mL IL21 (Peprotech #200-21), 10 µg/mL anti-IgM antibody (Southern Biotech #2020-01), and 2.5 µg/mL CpG-B (Hycult Tech #HC4039). For plasmablast differentiation, isolated naive B cells were cultured for 7 days in the presence of stimulating cocktail.

### Flow Cytometry Analysis

Isolated B cells were washed and stained with Live/Dead viability discrimination dye (Life Tech #L34968). Cells were subsequently blocked in 2% BSA supplemented with Fc Blocker (BioLegend) for 15 min and then stained with antibodies against B cell surface makers for 1 h [all antibodies were from BD Biosciences except CD38-PE/Texas Red (Life Technologies #MHCD3817); CD19-BV510, #562847; CD20-BUV396, #563782; CD27-BV421, #560448; IgD-APC, #348222; IgM-PerCP/Cy5.5, #314512; CD138-PE, #552026; IgG-PE/Cy7, #409316; CD45-APC/Cy7, #368516]. After staining, cells were washed two times in PBS without Mg^++^ or Ca^2+^ and then fixed in 2% PFA before analysis on a BD Fortessa or BD LSR flow cytometer. Plasmablasts were defined as CD19^+^CD20^+^IgD^−^CD27^+^CD38^+^ B cells. B cell activation was determined with CD86 surface staining (#562432). Ig production was determined by intracellular staining with anti-IgA (Life Tech #Z25002), anti-IgE (Biolegend #325510), and anti-IgG antibodies in plasmablasts; AID was detected with antibody #Z25302 (Life Tech). For intracellular IRF5 staining, after overnight fixation, cells were permeabilized the following day in 0.1% Triton X-100 and rinsed in PBS 2× before blocking in 2% BSA solution. IRF5 staining was performed using anti-IRF5 antibody conjugated to Alexa Fluor 488 (Abcam Catalog#: AB193245).

### IgG Isotype ELISA

Secretion of IgG isotypes was determined in media from 7-day cultures by ELISA (ThermoFisher #991000), as per manufacturer instructions.

### Proliferation Assay

Nucleofected cells were stained with 2.5 µM CFSE proliferation dye (Life Tech #C34554) or Cell Trace Violet dye (Life Tech #C34557) for 20 min. Cells were then washed, plated, and stimulated for 5 days. Proliferation was analyzed in Live/Dead^−^CD19^+^CD20^+^CD38^lo^ B cells.

### IRF5 ChIP-Seq

Primary naive B cells were isolated as previously outlined and plated at a density of 1 × 10^6^/mL. B cells were either mock or anti-IgM^+^ CpG-B stimulated for 4 h at 37°C in Iscove’s Modified Dulbecco’s Media (Thermo Fisher) supplemented with 10% FBS. Cells were subsequently washed 2× in PBS-Ca^2+^-Mg^2+^ and fixed in 1% methanol-free paraformaldehyde for 10 min at 37°C. The cross-linking reaction was quenched by the addition of glycine to a final concentration of 0.125 M. Cells were then washed 2× in PBS-Ca^2+^-Mg^2+^ and the final cell pellet lysed in 300 μL of RIPA lysis buffer. Lysates were loaded into a Covaris sonication microtube and sonicated in a Covaris S2 immersion sonicator with a duty cycle of 5%, intensity 2, at 200 burst for 15 cycles at 1 min each. Lysates were then precleared with 75 µL of a 50% agarose protein a/g bead slurry for 1 h. IRF5 immunoprecipitation (IP) was performed overnight at 4°C on precleared lyates using 4 µg of validated IRF5 antibody (Abcam#: ab124792) (34). A 40% protein a/g bead slurry was added to each IP for 3 h to allow conjugation of IRF5 antibody to beads. IP bead samples were then washed 3× in each of the following buffers in order—RIPA lysis buffer, LiCl wash buffer (100 mM Tris pH 7.5, 500 mM LiCl, 1% NP-40, 1% sodium deoxycholate), and TE buffer (10 mM Tris–HCl pH 7.5, 10 mM Na_2_EDTA). IP Beads were incubated overnight in elution buffer (1% SDS/0.1 M NaHCO_3_) at 65°C to reverse cross-links. Eluted DNA was then treated with RNAse (0.5 mg/mL) and Proteinase K (10 mg/mL) for 2 h each before isolation using phenol chloroform extraction. Briefly, an equal volume of phenol chloroform was added to each sample, followed by vigorous vortexing. The mixture was then transferred to a phase lock tube, spun for 5 min at max speed, and the aqueous phase removed. Repeated ethanol precipitation was used to isolate final DNA. Resulting ChIP DNA was quantified by bioanalyzer to ensure sufficient yield and proper fragmentation. Samples were submitted to the New York University (NYU) Genome Center for library preparation and single-end sequencing on Illumina HiSeq to a read depth of 50 million reads. Ramos B cell samples were submitted to the Rutgers NJMS Genomic Sequencing Core for ChIP-Seq.

### RNA-Seq of Primary Human B Cells

Isolated primary B cells nucleofected with mock, scrambled, or IRF5 siRNA were mock or anti-IgM^+^ CpG stimulated for 6 h at 37°C. RNA was purified using the Qiagen RNeasy isolation kit and on-column DNA digestion performed to remove genomic DNA. Final RNA was eluted in 30 µL RNAse-free water and submitted to the NYU Genome Center for single end sequencing on Illumina HiSeq to a depth of 20 million reads in the case of primary B cells and 40 million reads for Ramos B cells.

### Bioinformatics Analysis of ChIP-Seq and RNA-Seq Data

ChIP-Seq data was processed by HiChIP (37). Reads were mapped to the Hg19 human reference sequences using BWA at default parameters (38). Peaks were called using the MACS2 algorithm at the following parameters (*p* = 0.0001, *m* = 10.30) and using IgG isotype controls as input against IRF5 ChIP treated samples. Motif enrichment analysis was performed using the TomTom Motif Comparison suite (MEME Suite 4.8) (39). Peaks were visualized using IGV genome browser (40). RNA-Seq data QC, read mapping by STAR (41), read counting by featureCounts (42) were handled through the QuickRNASeq pipeline developed at Pfizer (43). Subsequently, EdgeR was used to determine differentially expressed genes; a *p*-value ≤ 0.05 and a false discovery rate (FDR) <0.05 after Benjamin-Hochberg correction was used for determining significant differential gene expression (44). Normalized RNA-Seq data are presented as reads per kilobase of transcript per million reads mapped (RPKM) (45). The QC report, processed read count table, RPKM table, and interactive data exploring tool generated by QuickRNAseq is available at https://baohongz.github.io/IRF5_knockdown. We used HOMER (46) for pathway analysis as it contains a program for performing functional enrichment analysis from a list of genes (http://homer.ucsd.edu/homer/microarray/go.html) HOMER uses a one-sided Fisher’s exact test to determine the significance of over-representation of a gene set in the input list. We focused our analysis on enriched pathway gene sets from WikiPathways (47) as it is the most comprehensive open source pathway collection. A more stringent cutoff of log2 fold-change ≥1 and FDR ≤ 0.001 was applied to select differentially expressed genes before performing the enrichment analysis to minimize the impact of false positives due to the small sample size.

### Statistical Analyses

Unless otherwise stated, one-way ANOVA or two-way ANOVA were used to compare means among three or more independent groups. Bonferroni posttest to compare all pairs of data sets was determined when overall *p*-value was <0.05. All statistical analyses were performed using GraphPad Prism (version 7.0). Data are reported as mean ± SD. In each figure legend, the number (n) of biological repeats included in the final statistical analysis is indicated. *p*-value < 0.05 was considered significant.

## Results

### TLR9/BCR Stimulation Induces IRF5 Nuclear Translocation

**Figure 1:**
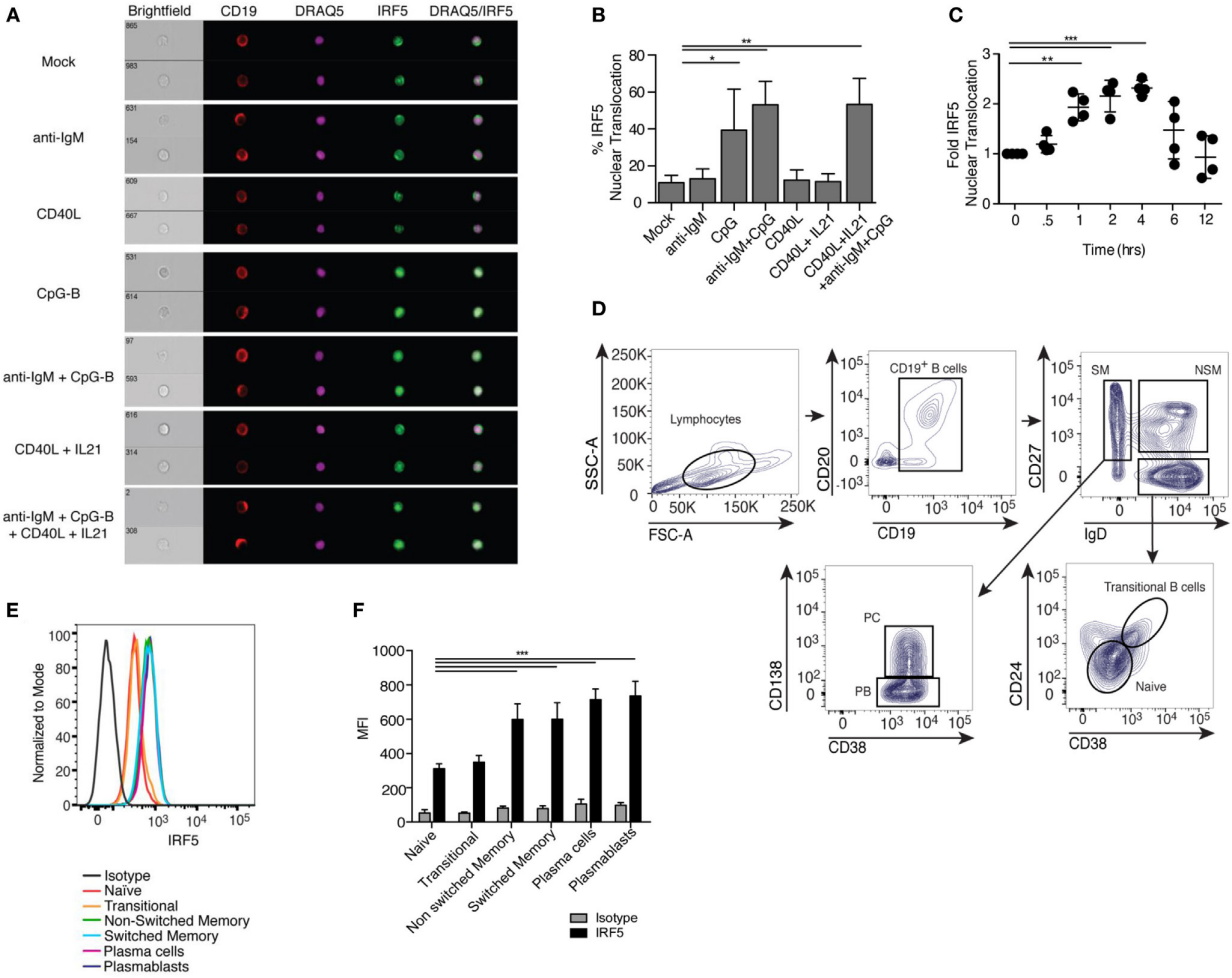
Toll-like receptor 9/B cell receptor stimulation induces Interferon regulatory factor 5 (IRF5) nuclear translocation. **(A)** Representative images of IRF5 cellular localization in human CD19^+^ B cells from a single healthy donor that were stimulated with mock, anti-immunoglobulin (Ig) M antibody, CD40 ligand (CD40L), CpG-B, or CD40L and IL21, and the combination of anti-IgM antibody, CD40L, IL21, and CpG-B for 2 h. PBMC were surface-stained with anti-CD19 antibodies, fixed and permeabilized, then stained for intracellular IRF5 and nuclear DRAQ5. Samples were then subjected to imaging flow cytometry followed by analysis in the IDEAS software suite. **(B)** Frequency of cells in **(A)** with IRF5 nuclear translocation, which was defined by an IRF5 and DRAQ5 similarity score ≥2 (one-way ANOVA with Tukey’s *post hoc* test; *n* = 4 independent donors). **(C)** IRF5 nuclear translocation was quantified over 12 h in isolated B cells following stimulation with anti-IgM^+^ CpG-B. Data was normalized to mock to minimize donor variability (one-way ANOVA with Tukey’s *post hoc* test; *n* = 4 independent donors). **(D)** Representative gating strategy for human B cells subsets in peripheral blood of healthy donors who received the influenza vaccine 7 days prior to phlebotomy. B cell populations were defined as CD19^+^CD20^+^ naive (IgD^+^CD38^−^CD27^−^CD24^−^), transitional (IgD^+^CD27^−^CD38^+^CD24^+^), non-switched memory (NSM; IgD^+^CD27^+^CD38^−^CD24^−^), switched memory (SM; IgD^−^CD27^+^CD24^−^), plasma blasts (PB; IgD^−^CD38^hi^CD27^+^CD24^−^CD138^−^), and plasma cells (PC; IgD^−^CD38^hi^CD27^+^CD24^−^CD138^+^). **(E)** Representative histograms of IRF5 protein expression in B cell subsets gated in (D). **(F)** Average MFI of IRF5 expression in gated B cell subsets (two-way ANOVA with Tukey’s multiple comparison *post hoc* test; *n* = 5 independent donors). Error bars represent SD. *p ≤ 0.05; **p ≤ 0.01; ***p ≤ 0.001.

Among the pathways known to play a role in ASC differentiation is TLR signaling (48). IRF5 acts downstream of TLRs in monocytes and dendritic cells, but characterization of IRF5 activation in human B cells has not been shown. IRF5 resides in the cytoplasm of unstimulated cells and upon activation translocates to the nucleus (49–51). We examined IRF5 activation following treatment of healthy CD19^+^ B cells with activating stimuli using imaging flow cytometry (gating in Figure S1B in Supplementary Material). We failed to detect significant IRF5 nuclear translocation with BCR activating anti-IgM antibody or T cell-dependent CD40L but detected significant activation with the TLR9 agonist CpG-B (Figures 1A,B). As the majority of peripheral B cells are antigen naive and express low levels of TLR9, we combined BCR stimulation, known to upregulate TLR9, with CpG-B and examined IRF5 activation (52). Anti-IgM plus CpG-B also provided a significant increase in IRF5 nuclear translocation over mock (Figures 1A,B). Since ASC differentiation can utilize T cell-mediated signals, we examined IRF5 activation following stimulation with CD40L and IL21. Although we did not detect an increase in IRF5 activation with CD40L and IL21, the combination of CD40L, anti-IgM, IL21, and CpG-B significantly increased IRF5 nuclear translocation to levels seen with anti-IgM and CpG-B (Figures 1A,B).

In murine monocytes, IRF5 nuclear translocation was shown to increase linearly over time following stimulation (24). We measured IRF5 activation kinetics in human B cells following anti-IgM^+^ CpG-B stimulation. Significant IRF5 nuclear translocation was first observed at 1 h, with peak translocation occurring at 4 h (Figure 1C). To determine if activation is a byproduct of increased expression, we measured IRF5 protein levels in total B cells following 2 h stimulation; no significant difference in IRF5 levels was detected (Figures S1C,D in Supplementary Material). Although IRF5 expression was unchanged at this early time point of activation, IRF5 expression across B cell subsets may differ and has not previously been measured. In monocytes, increased IRF5 expression is known to be deterministic of subset fate, with higher IRF5 levels seen in inflammatory M1 macrophages (33). To determine if IRF5 expression has similar traits in B cells, we quantified expression in B cell subsets from healthy donors. Routine vaccination is known to increase the occurrence of plasmablasts and plasma cells in the periphery 7 days post-immunization (53). We therefore utilized peripheral blood from individuals immunized with the flu vaccine to define IRF5 protein levels in CD19^+^CD20^+^ naive (IgD^+^CD38^−^CD27^−^CD24^−^), transitional (IgD^+^CD38^+^CD27^−^CD24^+^), non-switched memory (IgD^+^CD38^−^CD27^+^CD24^−^), switched memory (IgD^−^CD27^+^CD24^−^), plasmablasts (IgD^−^CD38^hi^CD27^+^CD24^−^CD138^−^), and plasma cells (IgD^−^CD38^hi^CD27^+^CD24^−^CD138^+^) (Figure 1D). Naive and transitional B cells expressed lower levels of IRF5 protein, whereas both non-switched and switched memory had increased expression. Plasmablasts and plasma cells had the highest IRF5 expression (Figures 1E,F). Similar findings were made in blood from healthy, non-vaccinated individuals (Figures S1E,F in Supplementary Material). These data suggest that IRF5 may regulate both B cell subset fate and effector function in mature B cell populations.

### IRF5 Knockdown Reduces ASC Differentiation

**Figure 2:**
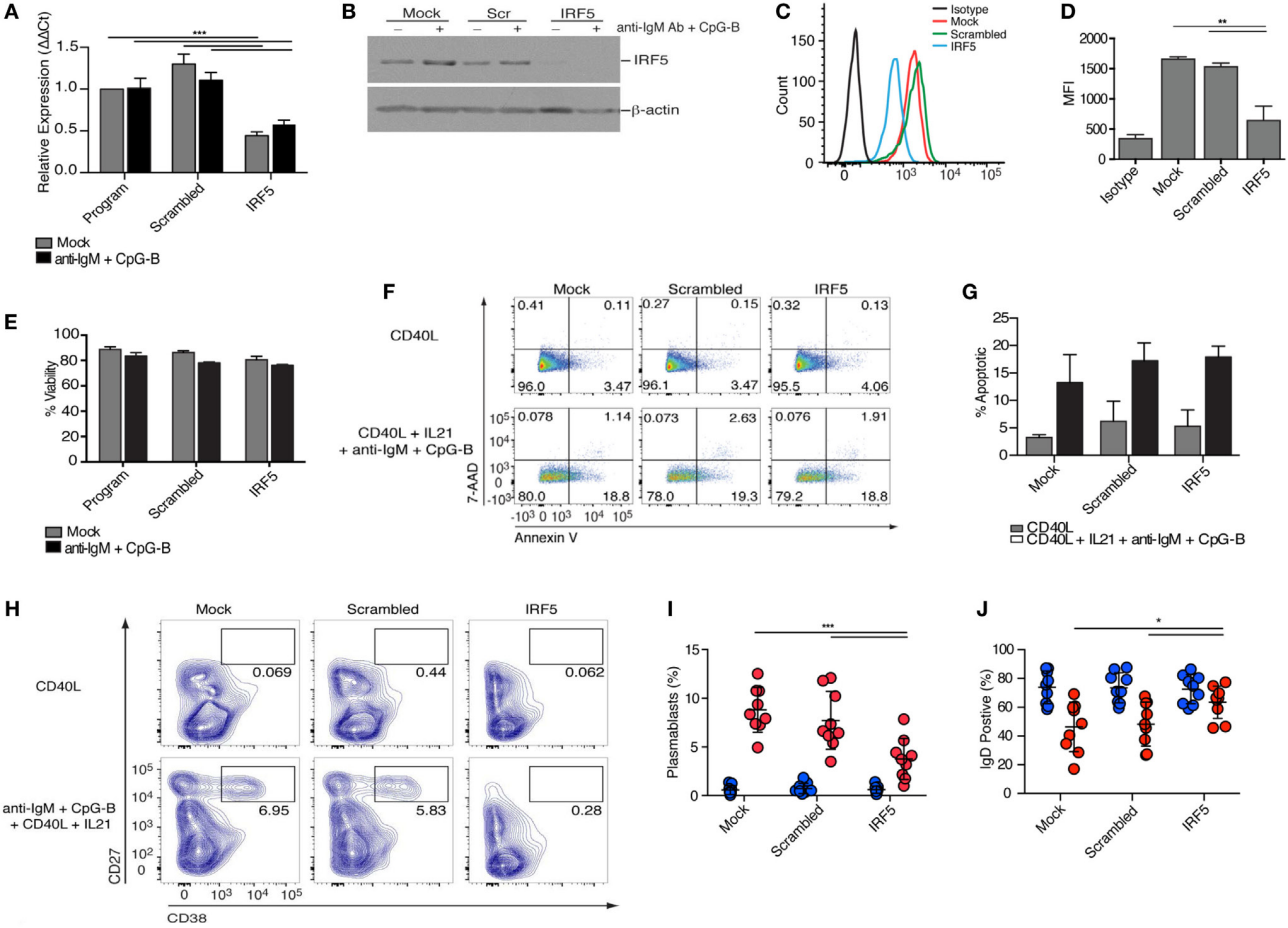
Interferon regulatory factor 5 (IRF5) is required for toll-like receptor 9/B cell receptor -induced antibody secreting cell differentiation. Isolated human naive B cells were nucleofected with 500 nM of mock, scrambled or IRF5 siRNA and mock-stimulated or stimulated with the indicated cocktails. **(A)** *IRF5* transcript expression was quantified through qPCR on RNA isolated 48 h postnucleofection and 12 h poststimulation with anti-IgM^+^ CpG-B (two-way ANOVA with Tukey’s *post hoc* test; *n* = 4 independent donors). **(B)** Protein lysates were prepared from nucleofected B cells 72 h postnucleofection and 24 h poststimulation with anti-IgM^+^ CpG-B. Western blot is one representative experiment out of three performed on *n* = 3 independent donors. **(C)** Representative histograms of IRF5 expression 48 h postnucleofection. Viable cells were analyzed through live/dead staining discrimination. **(D)** Plotted MFI of IRF5 from **(C)** (one-way ANOVA with Tukey’s *post hoc* test; *n* = 4 independent donors). **(E)** Plotted percentage of B cell viability assessed 72 h post-nucleofection, as determined through trypan blue exclusion. Data are from *n* = 3 independent donors. **(F)** Representative dot plots from B cell apoptosis quantified following staining with Annexin V and 7 amino-actinomycin D (7-AAD). Early apoptotic events are characterized as Annexin V^+^ 7AAD^−^, whereas late apoptotic events are Annexin V^+^ 7AAD^+^. Quantitation is shown in **(G)**. **(G)** Average apoptotic B cells from **(F)** 96 h post-nucleofection and 48 h post-stimulation (Two-way ANOVA with Tukey’s *post hoc* test; *n* = 3 independent donors). **(H)** Isolated naive B cells were nucleofected with 500 nM of mock, scrambled or IRF5 siRNA and stimulated with either CD40L or the combination of CD40L, IL21, anti-IgM, and CpG-B for 7 days. Plasmablast differentiation was quantified through gating of CD19^+^CD20^+^IgD^−^CD27^+^CD38^+^ B cells; final CD27^+^CD38^+^ gating is shown. Flow cytometry contour plots are representative of one experiment from a single donor. **(I)** Average number of plasmablasts from **(H)** following culture for 7 days (two-way ANOVA with Tukey’s *post hoc* test; *n* = 9 independent donors). **(J)** Average number of IgD^+^CD38^lo^ B cells from **(H)** following stimulation (two-way ANOVA with Tukey’s *post hoc* test; *n* = 9 independent donors). Error bars represent SD. *p ≤ 0.05; **p ≤ 0.01; ***p ≤ 0.001.

To investigate the role of IRF5 in human ASC differentiation, we validated and optimized targeted IRF5 knockdown in human primary naive B cells using nucleofection (36, 54, 55). Isolated naive B cells were nucleofected with mock, ON-TARGETplus non-targeting control pool (scrambled), or SMARTpool ON-TARGETplus human IRF5 siRNA. IRF5 knockdown efficiency was quantified by measuring IRF5 transcript and protein levels. A 50% reduction in *IRF5* transcript levels was detected 24 h postnucleofection in comparison to mock and scrambled (Figure 2A). To ensure that a reduction in transcripts correlated with a reduction in IRF5 protein levels, protein lysates were prepared at 72 h postnucleofection (with 24 h anti-IgM plus CpG-B stimulation) and analyzed by Western blot and flow cytometry (Figures 2B–D). Costimulation provided an increase in IRF5 expression levels over mock unstimulated, and these levels were significantly reduced following nucleofection with *IRF5* siRNA, while IRF5 levels remained steady in mock and scrambled controls supporting the specificity of knockdown. Notably, analysis by flow cytometry showed reduced IRF5 expression amongst the total population of viable B cells in comparison to scrambled control. Stimulation with anti-IgM and CpG-B did not impact IRF5 knockdown efficacy (Figures 2A,B).

Interferon regulatory factor 5 is known to regulate the transcription of pro-apoptotic genes following DNA damage or death receptor signaling (56, 57). To quantify B cell viability following IRF5 knockdown, nucleofected naive B cells were stained with tryphan blue 24 h after mock or anti-IgM and CpG-B stimulation. Although nucleofection itself results in ~15% reduction in cell viability, no further decrease in viability was found after IRF5 knockdown (Figure 2E). As viability assays often fail to detect early stages of apoptosis, we assayed B cell apoptosis following IRF5 knockdown through Annexin V and 7-amino-actinomycin D (7-AAD) staining. CD40L treatment did not induce significant levels of apoptosis, whereas stimulation with CD40L, IL21, anti-IgM, and CpG-B induced a similar increase in apoptosis across all three siRNA conditions (Figures 2F,G). Upon exposure to antigen, only a small proportion of naive B cells will be selected to undergo ASC differentiation, thus the observed increase in apoptosis by the combination treatment is not surprising. However, IRF5 knockdown did not result in a significant decrease in apoptosis in comparison to control conditions (Figure 2G). This indicates that under the current experimental conditions, IRF5 does not have a significant impact on B cell viability or apoptosis.

Following stimulation, B cells rapidly proliferate and differentiate to ASCs. In an effort to measure ASC generation from the population of naive B cells showing IRF5 knockdown, naive B cells were conucleofected with GFP mRNA and *IRF5* siRNA. Unfortunately, only ~20% of naive B cells expressed GFP and no correlation between GFP and IRF5 knockdown was found (Figures S2A–C in Supplementary Material). Similar effects were seen with pmaxGFP™; in this case, only ~2–4% of naive B cells expressed GFP (Figures S2D,E in Supplementary Material). Thus, cells were nucleofected as before and cultured for 2 days, followed by stimulation with CD40L or anti-IgM, CpG-B, CD40L, and IL21 for 7 days. These stimulation conditions result in a significant increase in IgD^−^CD27^+^CD38^+^ plasmablasts (6, 58), as seen in both mock and scrambled control (Figures 2H,I, flow gating in Figure S3A in Supplementary Material). In contrast to these controls, IRF5 knockdown resulted in a two-fold reduction in plasmablast differentiation. Additionally, the percentage of IgD^+^ B cells present following stimulation was significantly increased after IRF5 knockdown (Figure 2J; Figure S3B in Supplementary Material). Similar to that observed in the circulation of influenza-vaccinated healthy donors (Figures 1E,F), increased IRF5 expression was detected in B cells over the 7-day *in vitro* culture period. As expected, naive B cells expressed the lowest levels of IRF5 and plasmablasts expressed the highest (Figures S2F,G in Supplementary Material). Together, these data indicate that IRF5 has a B cell-intrinsic role in human ASC differentiation.

### IRF5 Knockdown Impairs B Cell Proliferation and Activation

**Figure 3:**
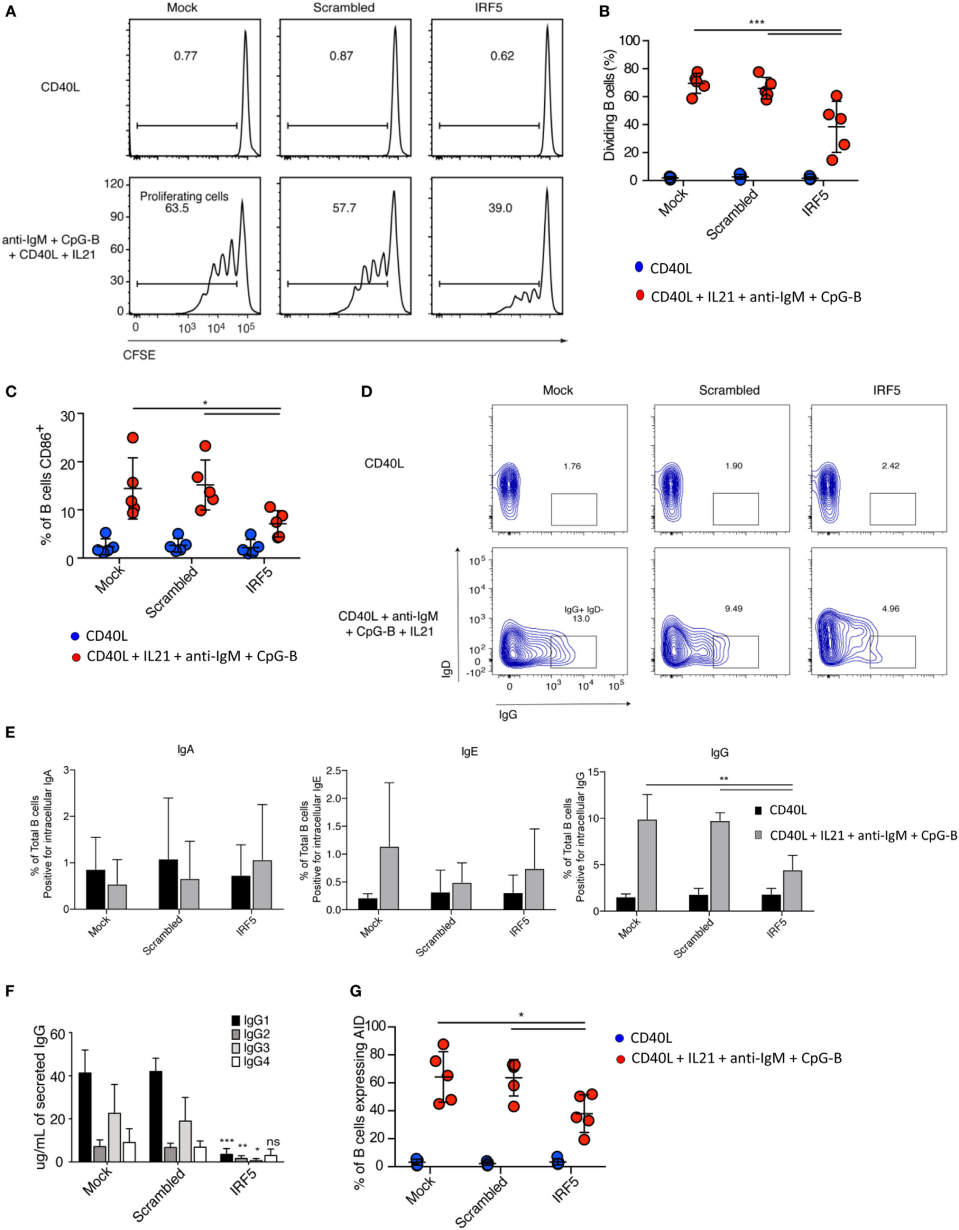
Interferon regulatory factor 5 (IRF5) knockdown impairs B cell proliferation, activation, and immunoglobulin (Ig) G isotype secretion. **(A)** Representative flow cytometry histograms from cell proliferation assay as measured through dilution of the proliferation dye CFSE. Isolated primary naive B cells from a single donor were nucleofected with 500 nM of mock, scrambled or IRF5 siRNA and stimulated with either CD40 ligand (CD40L) or the combination of CD40L, IL21, anti-IgM, and CpG-B for 5 days. **(B)** Average percentage of proliferating B cells from **(A)** is shown (two-way ANOVA with Tukey’s *post hoc* test; *n* = *5* independent donors). **(C)** Average percentage of B cells expressing CD86. Similar to **(A)** except isolated naive B cells were nucleofected and then stimulated with CD40L, IL21, anti-IgM, and CpG-B for 24 h, then stained for surface CD86 (two-way ANOVA with Tukey’s *post hoc* test; *n* = 5 independent donors). **(D)** Representative contour plots from IgD^−^IgG^+^ B cells after gating on total CD19^+^CD20^+^ B cells. Isolated primary naive B cells were nucleofected with 500 nM of mock, scrambled or IRF5 siRNA and stimulated with either CD40L or the combination of CD40L, IL21, anti-IgM, and CpG-B for 7 days. **(E)** Quantification from **(D)** of Ig antibody class expression was determined by intracellular flow cytometery (one-way ANOVA with Tukey’s *post hoc* test; *n* = 5 independent donors). **(F)** Cell culture supernatants from **(D)** were used for ELISA to determine IgG isotype secretion. Average concentration of IgG isotype is shown (two-way ANOVA with Tukey’s *post hoc* test; *n* = 4 independent donors). **(G)** Frequency of activation-induced deaminase (AID) expression in B cells following nucleofection with 500 nM of mock, scrambled or IRF5 siRNA and stimulated with CD40L, IL21, anti-IgM, and CpG-B for 3 days. AID expression was determined by intracellular flow cytometry (one-way ANOVA with Tukey’s *post hoc* test; *n* = 5 independent donors). Error bars represent SD. *p ≤ 0.05; **p ≤ 0.01; ***p ≤ 0.001.

Stimulation of B cells results in a rapid proliferative burst, which is required for sufficient numbers of antigen-specific B cells to survive CSR. To investigate if a decrease in the number of B cells entering proliferation is responsible for the reduction in plasmablasts seen after IRF5 knockdown, we assayed B cell proliferation. Nucleofected B cells were labeled with the proliferation dye CFSE, stimulated with anti-IgM, CpG-B, CD40L, and IL21 for 5 days, and CFSE dilution determined through flow cytometery by gating on viable cells (Figure S3A in Supplementary Material). A significant reduction in the number of naive B cells undergoing proliferation was seen following IRF5 knockdown (Figures 3A,B). Compared to mock and scrambled controls, IRF5 knockdown resulted in a twofold reduction in proliferation.

B cell activation occurs immediately following antigen recognition, and can occur through each of the previously described activation pathways. CpG-B stimulation of naive B cells can upregulate expression of the activation marker CD86 (6). As B cell activation primes for proliferation, we assayed activation following IRF5 knockdown. Primary naive B cells were nucleofected and stimulated for 2 days. Stimulation induced the upregulation of CD86 expression in both mock- and scramble-nucleofected B cells, whereas IRF5 knockdown significantly reduced CD86 expression (Figure 3C). IRF5 is known to function downstream of TLR9, which has previously been shown to be involved in the upregulation of CD86 (52, 58). These data indicate that IRF5 acts downstream of TLR9/BCR to induce early B cell activation and proliferation.

### IRF5 Knockdown Reduces Human IgG1 and 3 Secretion

Given the reduction in plasmablast differentiation seen after IRF5 knockdown, we determined whether there would be a consequential reduction in secreted antibody. We first measured percentages of nucleofected B cells expressing intracellular IgA, IgE, or IgG following stimulation for 5 days using flow cytometry. Intracellular Ig staining is indicative of the fraction of B cells that may secrete a specific antibody isotype. Representative flow gating for IgG in total CD19^+^CD20^+^ B cells is shown in Figure 3D. While few B cells expressed intracellular IgA or IgE, a large fraction of B cells stained positive for IgG. IRF5 knockdown resulted in a significant reduction in intracellular IgG staining (Figure 3E). IRF5 has previously been linked to IgG isotype secretion in mice, with *Irf5^−/−^* mice having reduced IgG2a levels following viral infection and in murine models of lupus (21, 22). As IL21 and CpG have both been shown to predominantly promote IgG secretion (59), we further measured IgG isotypes by ELISA. Following stimulation, IRF5 knockdown resulted in significantly decreased levels of IgG1, 2, and 3 but not 4 (Figure 3F). Since IgG2 and 4 were only secreted to a low extent, the largest impact was among the IgG1 and 3 isotypes. To determine if the reduction in IgG expression was due to a reduction in CSR, we assayed AID expression. AID expression is transcriptionally regulated, with expression quickly upregulated following B cell activation and proliferation. Interestingly, expression of AID was significantly decreased following IRF5 knockdown (Figure 3G). These data indicate that IRF5 contributes to early stages of ASC differentiation.

### Identification of New IRF5 Target Genes Associated with ASC Differentiation

**Figure 4:**
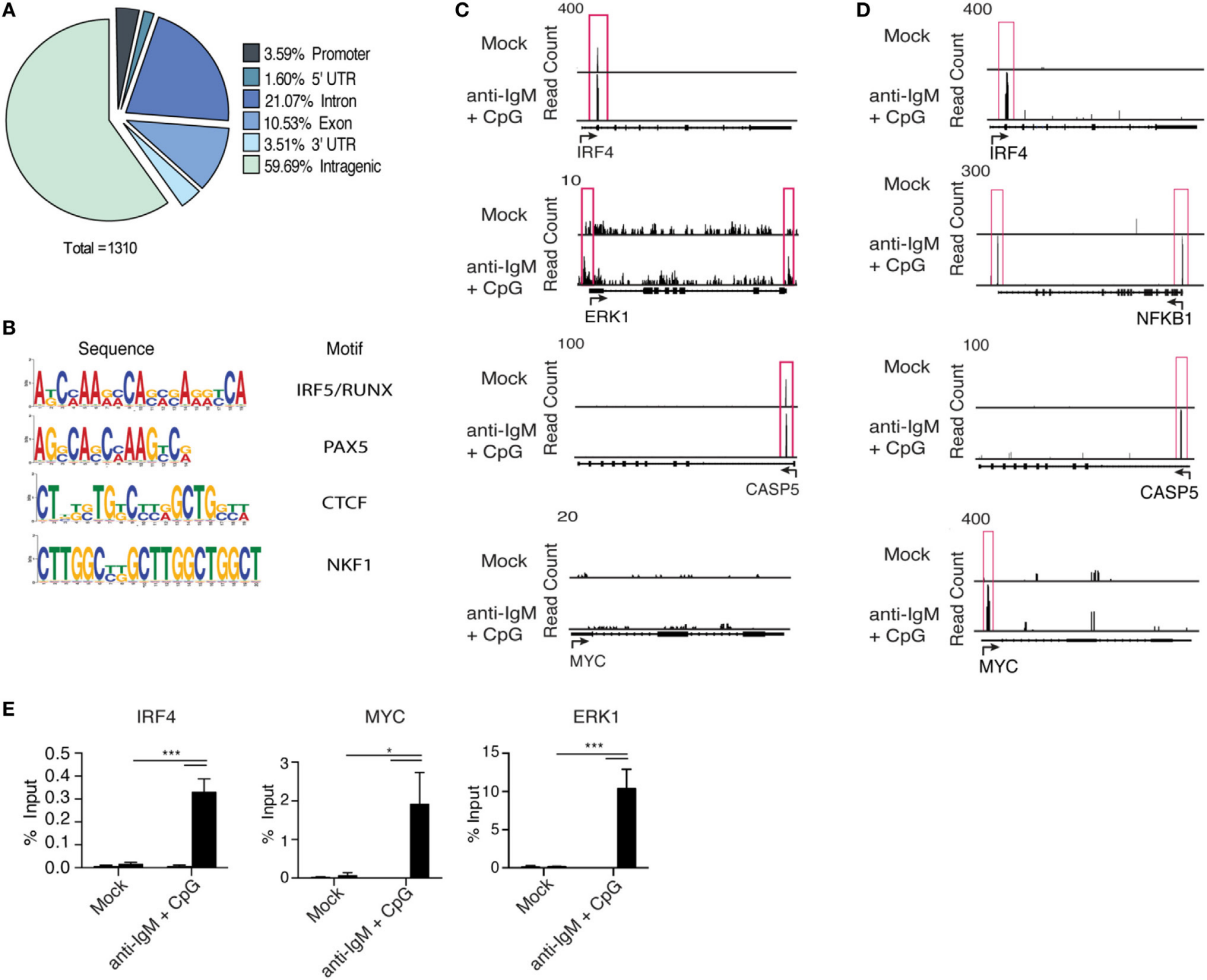
Interferon regulatory factor 5 (IRF5) binds promoter regions of genes associated with antibody secreting cell (ASC) differentiation. IRF5 ChIP-Seq was performed on isolated primary naive B cells from *n* = 2 independent donors. B cells were either mock or anti-IgM^+^ CpG-B stimulated for 4 h. Reads were mapped through BWA and peaks called through MACs. **(A)** A pie chart showing representative IRF5 binding elements throughout the human primary B cell genome. **(B)** Common IRF5 binding motifs identified from ChIP-Seq. Motif sequences are shown in order of enrichment with associated transcription factor motif. **(C)** Representative peak distributions are shown for *IRF4, ERK1, CASP5*, and *MYC*. Peaks are boxed in red and were determined at a significance of *p* ≤ 0.0001. **(D)** Same as **(C)** except IRF5 ChIP-Seq peaks are from the Ramos B cell line. Peaks were determined at *p* ≤ 10^−10^. **(E)** Independent confirmation of IRF5 binding to *IRF4, MYC*, and *ERK1* target sites through ChIP-qPCR in primary naive B cells mock stimulated or stimulated with anti-IgM^+^ CpG-B for 4 h (two-way ANOVA with Tukey’s *post hoc* test; *n* = 4 independent donors). Error bars represent SD. *p ≤ 0.05; ***p ≤ 0.001.

Interferon regulatory factor 5 initiates transcription of target genes following nuclear translocation; however, IRF5 target genes in B cells have not been characterized. We performed chromatin IP combined with deep sequencing (ChIP-Seq) to identify IRF5 transcriptional targets across the genome in human primary naive B cells. To ensure IRF5 is specifically enriched in ChIP experiments, previously validated antibodies were used for IP (36). ChIP-Seq libraries were prepared from both mock and anti-IgM^+^ CpG-B-stimulated cells (4 h) to characterize activation-dependent targets of IRF5. Computational analysis revealed a sharp increase in the number of IRF5 peaks following stimulation. In mock-treated samples, IRF5 bound 22 genes, and upon treatment with anti-IgM^+^ CpG-B, IRF5 targets increased to 784. IRF5 genome occupancy was distributed across various gene elements with 3.5% of peaks occurring within ±3 kb of putative transcription start sites (Figure 4A). Additionally, a large majority of peaks were found on intragenic regions. Roughly 10.5% of peaks were bound to exons, and 21% were bound to non-coding regions.

Interferon regulatory factor family members bind to IFN-stimulated response elements (ISREs) consisting of the *GAANNGAA* motif. IRF5, however, can partner with other transcription factors to recognize various consensus sequences. It was recently reported that IRF5, in cooperation with RelA of the NFĸB pathway, can recognize composite PU.1/ISRE motifs in murine inflammatory monocytes (60). IRF5 consensus sites have not been well-defined in humans. We therefore sought to determine if IRF5 recognizes similar motifs in human B cells. Using motif discovery algorithms, we identified common motifs within 100 bp of peak centers. Among the represented motifs was the expected ISRE consensus sequence, as well as a newly identified IRF5/RUNX motif that was equally enriched for (Figure 4B). These data suggest cooperation between IRF5 and RUNX family members in the targeting of genes following B cell activation.

Interferon regulatory factor 5 was found to bind a wide range of genes, including several associated directly with ASC differentiation. Among these targets were *IRF4, ERK1*, and *CASP5* (Figure 4C). While these genes are implicated in ASC differentiation and were targeted by IRF5 in human primary B cells following activation, none seemed to fully account for the early defects in B cell proliferation or activation (Figures 3A–C). As a result, there remained the possibility that several target genes were missing due to limitations, possibly, in obtaining sufficient numbers of primary naive B cells at the 4 h time point of IRF5 activation (Figure 1C). To ensure the identification of all possible IRF5 target genes, we performed IRF5 ChIP-Seq in Ramos B cells. Ramos are known to express high levels of TLR9, and thus recapitulate early stages of primary naive B cell activation following anti-IgM^+^ CpG-B stimulation (61). Enriched reads from Ramos B cells were similarly distributed as in primary B cells (Figure S4A in Supplementary Material). Importantly, identical target genes, such as *IRF4* and *CASP5*, were identified in both datasets (Figures 4C,D). Several additional target genes not seen in primary B cells were identified in Ramos. Among these was the proliferation gene *MYC*, and *NFKB1* (Figure 4D). To confirm that results from Ramos B cells were applicable to primary B cells, IRF5 ChIP-qPCR was performed on pooled primary naive B cells. Significant enrichment was seen on *IRF4, MYC*, and *ERK1* following IRF5 ChIP (Figure 4E). Interestingly, *MYC* is downstream of ERK1 and an ERK1-Elk-Myc signaling pathway has been implicated in early B cell proliferation (8, 62). These data support that IRF5 directly binds to promoters of genes important for early stages of B cell activation and proliferation.

### Identification of an IRF5-Dependent B Cell Transcriptome

**Figure 5:**
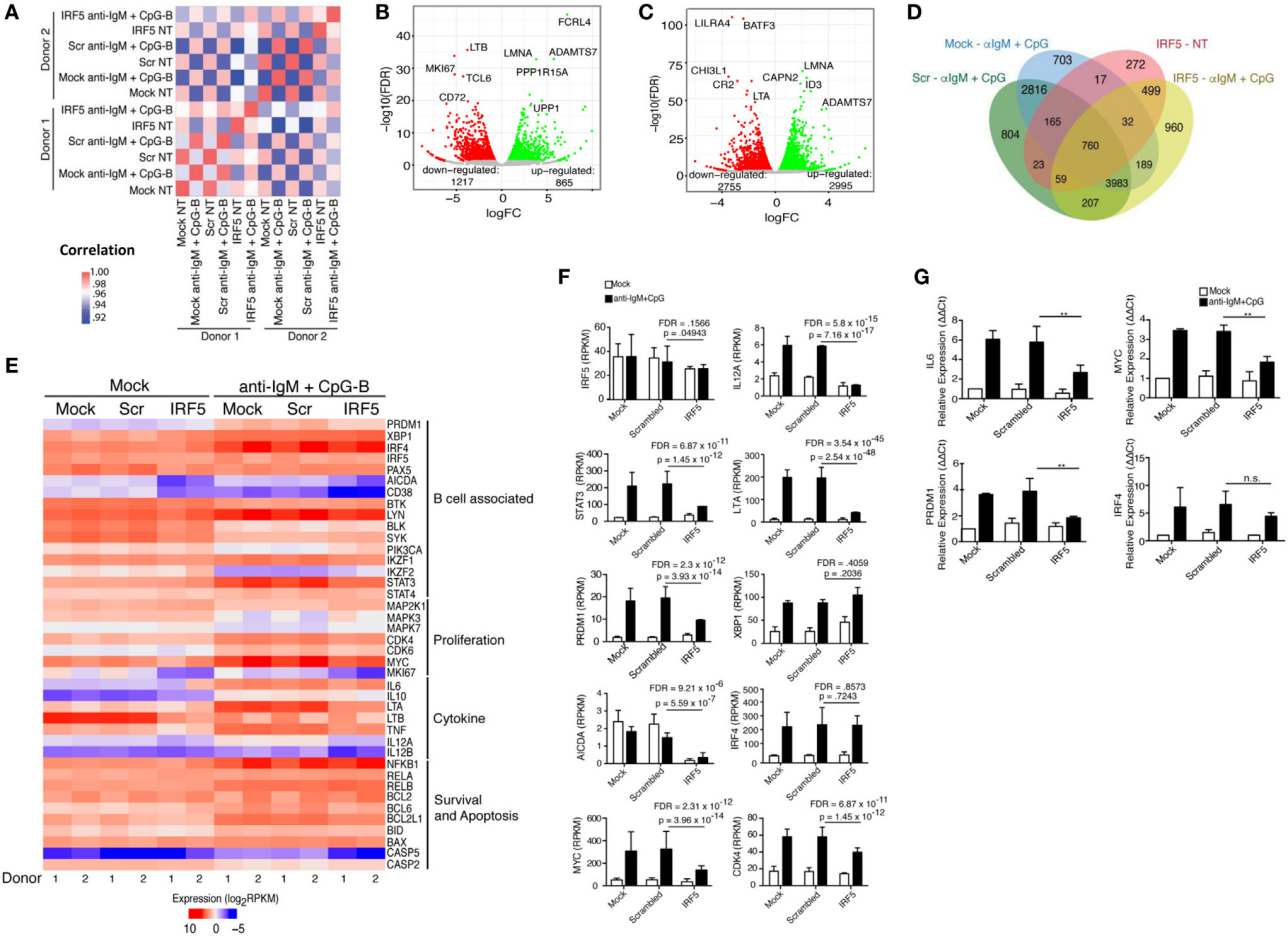
Identification of an interferon regulatory factor 5 (IRF5)-dependent B cell transcriptome. Isolated naive B cells were nucleofected with 500 nM of mock, scrambled or IRF5 siRNA and subsequently stimulated with anti-IgM and CpG for 6 h. RNA-seq was performed on B cells from *n* = 2 independent donors. **(A)** Significant donor correlation associated with IRF5 knockdown and stimulation. Heat map illustrates correlation coefficients for gene expression values between conditions (nucleofection and stimulation) and individual healthy donors. A strong correlation between samples is indicated by the red and pink boxes. Color legend is shown to the right indicating correlation coefficients. **(B)** Identification of genes with differential expression following IRF5 knockdown in mock-treated samples. Red circles indicate downregulated genes; green circles indicate upregulated genes. Differential gene expression was determined in comparison to scrambled control. **(C)** Same as **(B)** except genes were identified following IRF5 knockdown and stimulation (CpG-B^+^ anti-IgM). Differential expression was determined by comparison to stimulated scrambled control. **(D)** Diagram of differential expression between nucleofection and various stimulation conditions. Overlapping regions represent shared expression; single regions represent uniquely expressed genes. **(E)** Heat map of gene expression between the two donors based on cellular function associated with B cell activation, proliferation, and antibody secreting cell differentiation. Data are expressed as log_2_RPKM. **(F)** Raw RPKM values of particular genes relevant to IRF5 function. *p* values and false discovery rate (FDR) scores were obtained by multiple comparisons testing of samples indicated by line. Individual *p* values and FDR scores are included in each graph. **(G)** Independent confirmation of differential gene expression through qPCR (two-way ANOVA with Tukey’s *post hoc* test; *n* = 3 independent donors). Error bars represent SD. **p ≤ 0.01.

Identification of IRF5 target genes suggested that IRF5 could influence the transcription of several genes important for ASC differentiation. We thus examined changes on the B cell transcriptome following IRF5 knockdown by high throughput cDNA sequencing (RNA-Seq). Nucleofected B cells were mock or anti-IgM^+^ CpG-B stimulated for 6 h; the 6 h timepoint was chosen to account for early IRF5-mediated gene transcription occurring in response to nuclear IRF5 at 4 h poststimulation (Figure 1C). RNA-Seq was performed on two independent biological replicates showing a strong correlation coefficient between gene expression (Figure 5A). Bioinformatics analysis found a dramatic shift in the transcriptome of B cells following knockdown (Figures S4B,C in Supplementary Material). After knockdown in mock-treated cells, 1,217 genes were downgregulated while 865 genes were upregulated (Figure 5B). The most differentially expressed genes following IRF5 knockdown in the mock-treated group are listed in Table 1 (and shown in Figure S4B in Supplementary Material). Genes such as *FcRL4* and *SLC3A2*, which were significantly upregulated following IRF5 knockdown, have been shown to regulate B cell activation and proliferation. *FcRL4* is known to act as an inhibitor of BCR signaling (63). *SLC3A2* was shown to be important for B cell proliferation and plasmablast differentiation in mice (64). Alternatively, the B cell cytokine *LTB*, as well as the proliferation marker *Ki67* were found to be significantly downregulated after IRF5 knockdown (65). Following stimulation, IRF5 knockdown resulted in 2,755 downregulated and 2,995 upregulated genes (Figure 5C). Among the most differentially expressed genes within this group (Figure S4C in Supplementary Material; Table 2), which are relevant to B cell activation, included the upregulated genes *ID3* and *NR4A1* (66–68). Both have been shown to be involved in B cell activation and proliferation. In contrast, the B cell-associated cytokine *LTA* and the proliferation marker *Ki67* were found both to be significantly downregulated.

**Table 1:** List of top differentially expressed genes comparing mock-treated with scrambled siRNA against IRF5 knockdown.^a^

Upregulated genes in mock-treated cells	
**Gene^b^**	**Function**
*FCRL4*	Member of the Ig receptor superfamily; acts as an inhibitor of B cell activation
*ADAMTS7*	Plays a role in activation of vascular smooth muscle cells. Unknown role in B cells
*LMNA*	Lamin A/C, major component of the nuclear lamina. Important in maintaining integrity of nuclear structure; involved in chromosome segregation during mitosis
*PPP1R15A*	Recruits protein phosphatase 1, which suppresses stress kinase-induced protein expression. Expression upregulated during cellular stress or DNA damage
*AMICA1*	Adhesion molecule expressed on cell surface; primarily on T cells. Function in B cells remains unclear
*CD109*	A glycosyl phosphatidylinositol (GPI)-linked glycoprotein that localizes to the cell surface. Acts as a TGF-b coreceptor which stimulates internalization and degradation of TFG-b receptors
*UPP1*	Catalyzes cleavage of uridine and deoxy-uridine to uracil and ribose- or deoxyribose-1-phosphate
*SLC3A2*	Cell surface transmembrane protein which makes up the heavy chain of the amino acid transporter CD98. Acts as an integrin binding protein. Shown to be required for B cell proliferation and antibody responses
*SQSTM1*	Sequestosome 1, a multifunctional signaling protein known to be an autophagy target as well as an ubiquitin binding scaffolding protein involved in NFkB activation through interaction with TRAF6
**Downregulated genes in mock-treated cells**	
*LTB*	Lymphotoxin beta, membrane protein of the TNF family. Inflammatory B cell cytokine
*CHI3L1*	Member of chitanase family of proteins; lacks chitanse activity. Unclear role in B cells
*MKI67*	Ki67, surfactant required for chromosome segregation in proliferating cells. Used as marker of proliferating cells
*TCL6*	Non-coding RNA of unknown function
*CPNE5*	Copine 5, calcium binding protein involved in regulating cell signaling
*TRIB2*	Member of tribble family of serine/threonine kinases. Regulates TLR5-mediated NFkB activation
*CD72*	Membrane protein containing ITIM motifs and C-type lectin domains. Negative regulator of BCR-mediated signaling by suppression of calcium mobilization. Recently shown to regulate B cell autoreactivity following TLR7 ligation with autoantigen Sm/RNP
*CORO2B*	Coronin 2B protein coding gene of unknown function
*FAM129C*	B cell novel protein 1, unknown signaling protein

*^a^Comparing gene expression between scrambled siRNA and IRF5 siRNA nucleofected samples*.

*^b^p–Value ≤0.05 and an FDR < 0.05 after Benjamin–Hochberg correction was used for determining significant differential gene expression. Genes are from Figure 5*.

**Table 2:** List of top differentially expressed genes comparing anti-IgM^+^ CpG-B stimulated with scrambled siRNA against IRF5 knockdown.^a^

Upregulated genes in anti-IgM^+^ CpG-B-treated cells	
*NR4A1*	Hormone nuclear receptor and transcription factor. Involved in cell cycle progression and regulation of apoptosis. NR4A1(Nur77)-deficient mice display increased B cell survival among CD38^+^ B cells. Increased autoantibody production also observed
*LMNA*	See above
*ID3*	Transcriptional regulator of basic helix-loop-helix family of transcription factors. Implicated in cell growth, apoptosis, and differentiation. Required for germinal center formation but acts as a negative regulator of plasma cell differentiation
*LPXN*	Leupaxin, a negative regulator of paxilin signaling. Negative regulator of BCR signaling
*NR4A2*	Hormone nuclear receptor and transcription factor
*CAPN2*	Calpain 2, an intracellular cysteine protease. Regulates cell migration; is a Pax5 target gene in B cells
*ADAMTS7*	See above
*ARL4C*	Small GTP binding protein and member of ADP-ribosylation factor family. Regulates microtubule dependent intracellular vesicular transport
*FLNA*	Filamin A, an actin binding protein responsible for cross-linking of actin filaments. Plays a role in regulating cell shape and structure
**Downregulated genes in anti-IgM^+^ CpG-B-treated cells**	
*LILRA4*	Ig-like receptor expressed on cell surface. Receptor for bone marrow stromal cell antigen-2; may be involved in regulating early B cell development
*BATF3*	Basic Leucine Zipper transcription factor within the AP-1 family of transcription factors. Interacts with Jun as a transcriptional regulator in several immune cell populations
*CHI3L1*	See above
*HAPLN3*	Member of hyaluronan and proteoglycan binding link protein gene family; major component of extracelluar matrix. Recently shown to be upregulated in IFNb-stimulated B cells
*MYBL2*	Member of MYB transcription factor family; regulates cyclin D1 expression. Regulates cell cycle progression. Highly expressed during early B cell development
*GCA*	Grancalcin, a calcium binding protein that interacts with TLR9 to mediate IRF7- and NFkB-dependent type 1 interferon expression
*SLC2A5*	Acts as a fructose transporter. Unclear role in B cells
*CR2*	Complement receptor 2; binds complement factor C3Dd. Involved in B cell activation
*LTA*	Lymphotoxin alpha; member of TNF family of cytokines. Mediates inflammatory and cytotoxic effects on various target cells

*^a^Comparing gene expression between scrambled siRNA and IRF5 siRNA nucleofected samples*.

*^b^p–Value ≤0.05 and an FDR < 0.05 after Benjamin–Hochberg correction was used for determining significant differential gene expression. Genes are from Figure 5*.

Differential expression contrasts between stimulated mock-, scrambled-, and IRF5-nucleofected samples identified several genes uniquely modulated following IRF5 knockdown (Figure 5D). While several of these genes showed significant differential expression and have been previously implicated in B cell biology, they failed to intuitively explain the observed reduction in B cell proliferation and differentiation. As a result, we performed a further gene expression analyses focused on genes relevant to B cell activation, proliferation, and ASC differentiation (Figure 5E). Confirmation of our stimulation conditions was seen through the significant upregulation of genes involved in ASC differentiation (*IRF4, XBP1, PRDM1*), inflammatory cytokine expression (*LTA, LTB*), and proliferation (*MYC, CDK4*) (Figure 5E). Analysis of differential expression following IRF5 knockdown revealed a significant decrease in many of the genes (Figures 5E,F). As expected, expression of inflammatory cytokines such as *IL6, LTA, LTB*, and *IL12A* was significantly reduced following knockdown. Among the genes known to regulate ASC differentiation, expression of *PRDM1, AICDA*, and *MYC* were significantly downregulated whereas expression of *IRF4* and *XBP1* were not significantly impacted by IRF5 knockdown at this time point (Figure 5F). This may suggest that while IRF5 binds to the *IRF4* promoter, it is dispensable in *IRF4* transcriptional regulation. In contrast, IRF5 occupancy was found on the promoter region of *MYC*, and a corresponding significant decrease in *MYC* gene expression was seen after knockdown (Figure 5F). Reduction in *MYC*, and other genes identified by RNA-Seq, was confirmed by real-time qPCR in independent donors (Figure 5G). We next examined genes identified in Figure 5E for IRF-E or ISRE consensus binding sites in their 5′- and/or 3′-UTR. Results in Table 3 reveal that while many of the genes do contain consensus binding sites that IRF5 may recognize, not all do. Instead, IRF5 may utilize non-classical sequences, such as those identified in Figure 4B for direct regulation. Altogether, data indicate that one mechanism by which IRF5 regulates human ASC differentiation is through the early control of B cell proliferation *via* direct regulation of *MYC* expression followed by alterations in the expression of other ASC-associated genes.

**Table 3:** List of ASC-associated genes from RNA-Seq that contain potential ISRE and/or IRF-E sites in their regulatory regions.^a^

Gene	Chromosome position (Hg19)	ISRE	IRF-E
*PRDM1*	chr6:106,534,195-106,557,814	106566840, 106547222	106562388
*XBP1*	chr22:29,190,548-29,196,560	ND	ND
*IRF4*	chr6:391,739-411,443	391724	409414
*IRF5*	chr7:128,577,994-128,590,088	128580725	128594612
*PAX5*	chr9:36,838,531-37,034,476	ND	36867441, 36868076, 37197361
*AICDA*	chr12:8,754,762-8,765,442	ND	ND
*CD38*	chr4:15,779,931-15,850,706	ND	ND
*BTK*	chrX:100,604,435-100,641,212	ND	100668558
*LYN*	chr8:56,792,386-56,925,006	56842848, 56863323	6773360, 56793604
*BLK*	chr8:11,351,521-11,422,108	ND	11349703
*IKZF1*	chr7:50,344,378-50,472,798	50340355, 50484586	50424402, 50444411, 50484587
*IKZF2*	chr2:213,864,411-214,015,058	213831888, 214030738, 214014429, 214017464, 214030738	213936673, 214015411, 214017463
*STAT3*	chr17:40,465,343-40,540,513	40460528	ND
*STAT4*	chr2:191,894,302-192,016,322	ND	191885173, 191964289
*MYC*	chr8:128,748,315-128,753,680	ND	ND
*MKI67*	chr10:129,894,925-129,924,468	ND	ND
*IL6*	chr7:22,766,766-22,771,621	ND	22763062, 22765259
*IL12A*	chr3:159,706,623-159,713,806	ND	ND
*NFKB1*	chr4:103,422,486-103,538,459	103424172, 103560981	103448890, 103459016, 103533964
*RELA*	chr11:65,421,067-65,430,443	ND	65431520
*RELB*	chr19:45,504,707-45,541,456	ND	ND
*CASP5*	chr11:104,864,967-104,893,895	ND	ND

*^a^Consensus sequences were identified through UCSC using Hg19 and transcription factor binding sites that are within 4 kb upstream of the transcription start site (TSS) and 4 kb downstream of the stop codon*.

*ND, not detected*.

**Figure 6:**
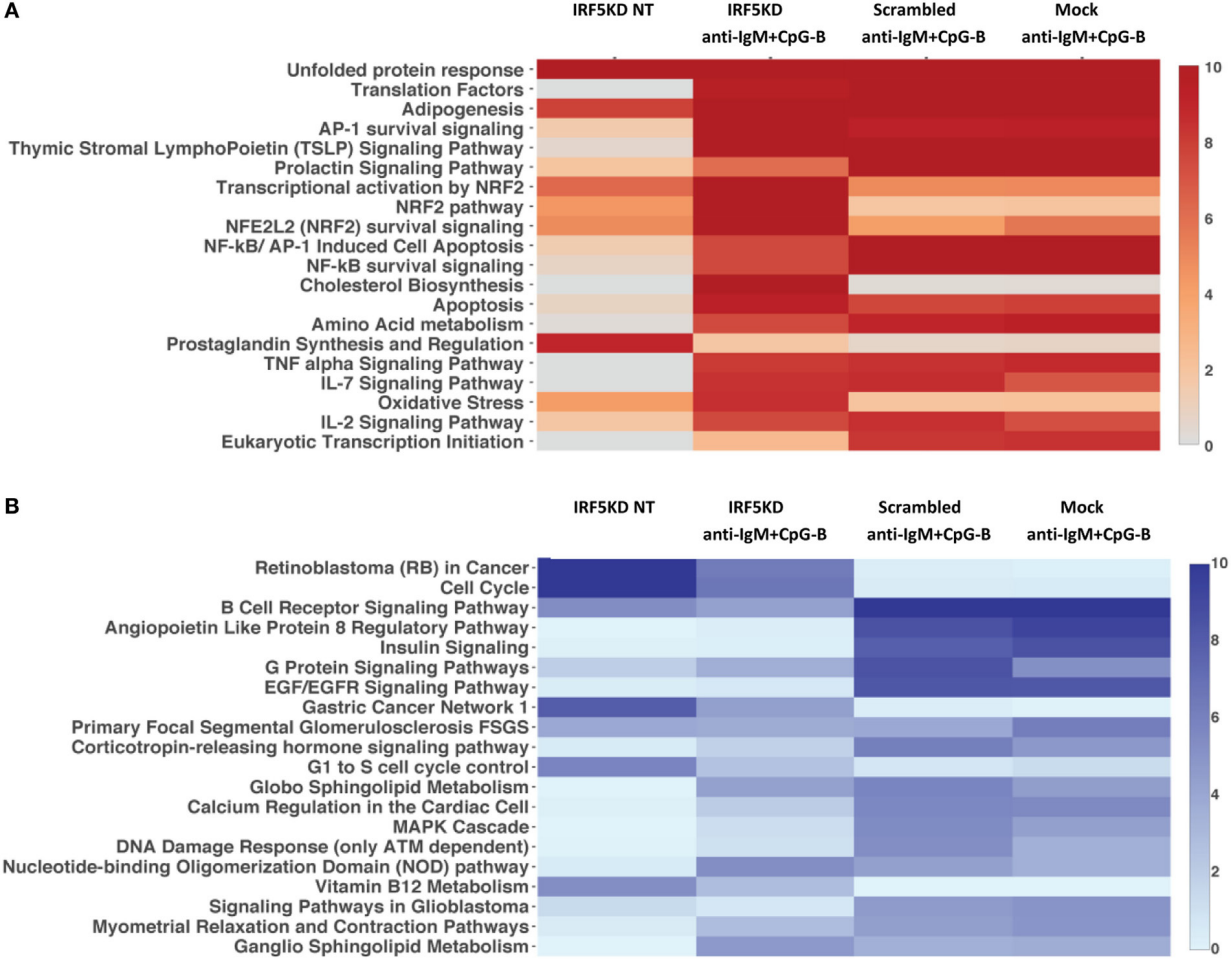

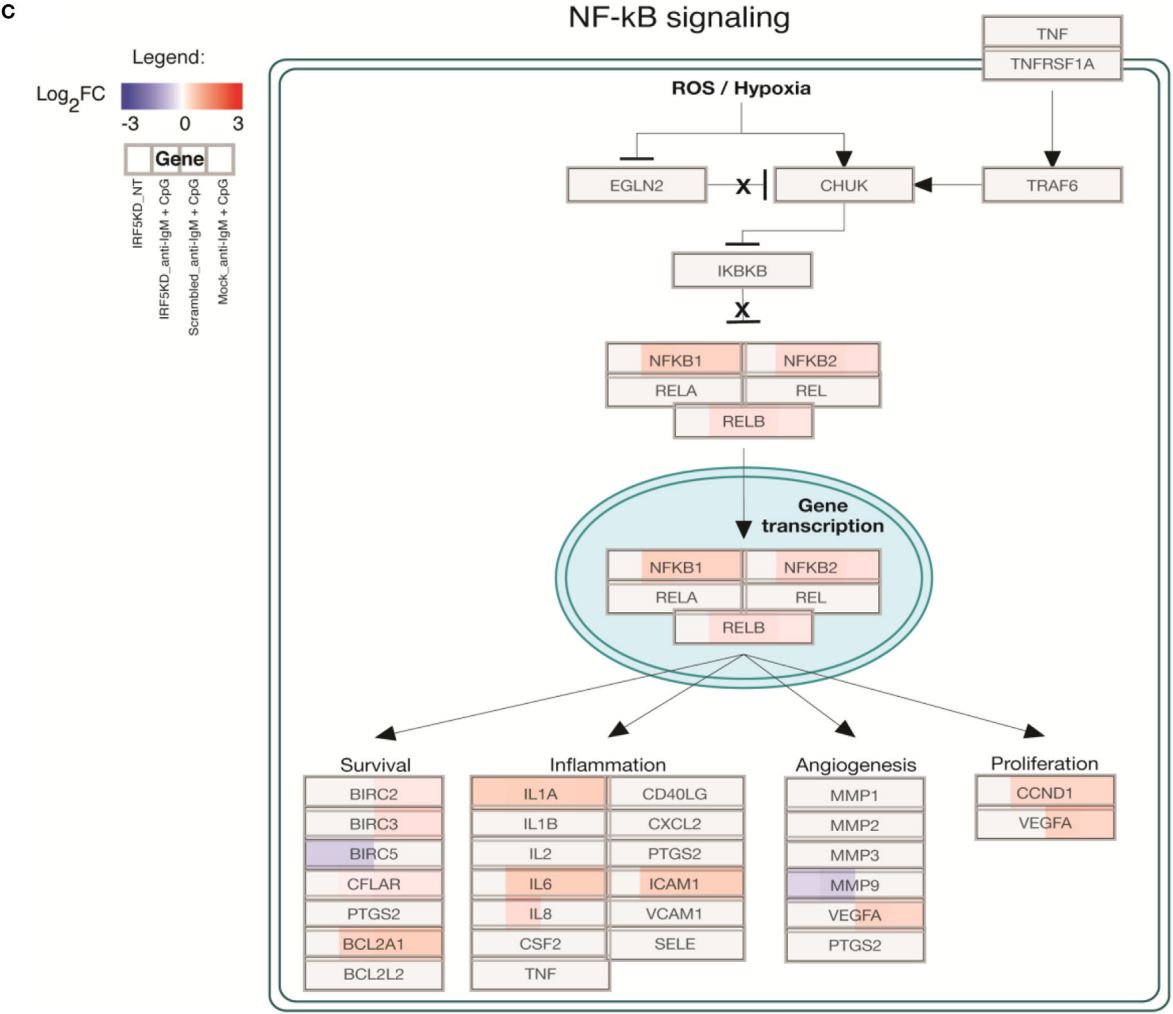
Interferon regulatory factor 5 (IRF5) regulates numerous biologic signaling pathways in human primary B cells. **(A)** Data from ChIP-Seq and RNA-Seq were used to identify the top 20 upregulated pathways in which there were more upregulated genes than downregulated genes as compared to Mock untreated (NT). Heat map is shown from HOMER analysis with a cutoff of log_2_FC ≥ 1 representing genes with at least a twofold change in expression and an false discovery rate (FDR) ≤ 0.001. Pathways are ordered by negative log10 *p*-values of enrichment. **(B)** Same as **(A)** except top 20 downregulated pathways are shown. **(C)** Representative scheme showing enrichment of the upregulated NF-ĸB survival signaling pathway after IRF5 knockdown. Boxes representing genes are color-coded based on the log2 fold-change values. Upregulated (red) or downregulated (blue) genes are shown as compared to Mock NT. Expression is shown by color code in four sections of each gene box. The gene box is divided into sections with one to one mapping comparisons of IRF5KD_NT, IRF5KD_anti-IgM^+^ CpG, Scr_anti-IgM^+^ CpG, and Mock_anti-IgM^+^ CpG vs. reference Mock_NT, as shown in the Legend.

**Figure 7:**
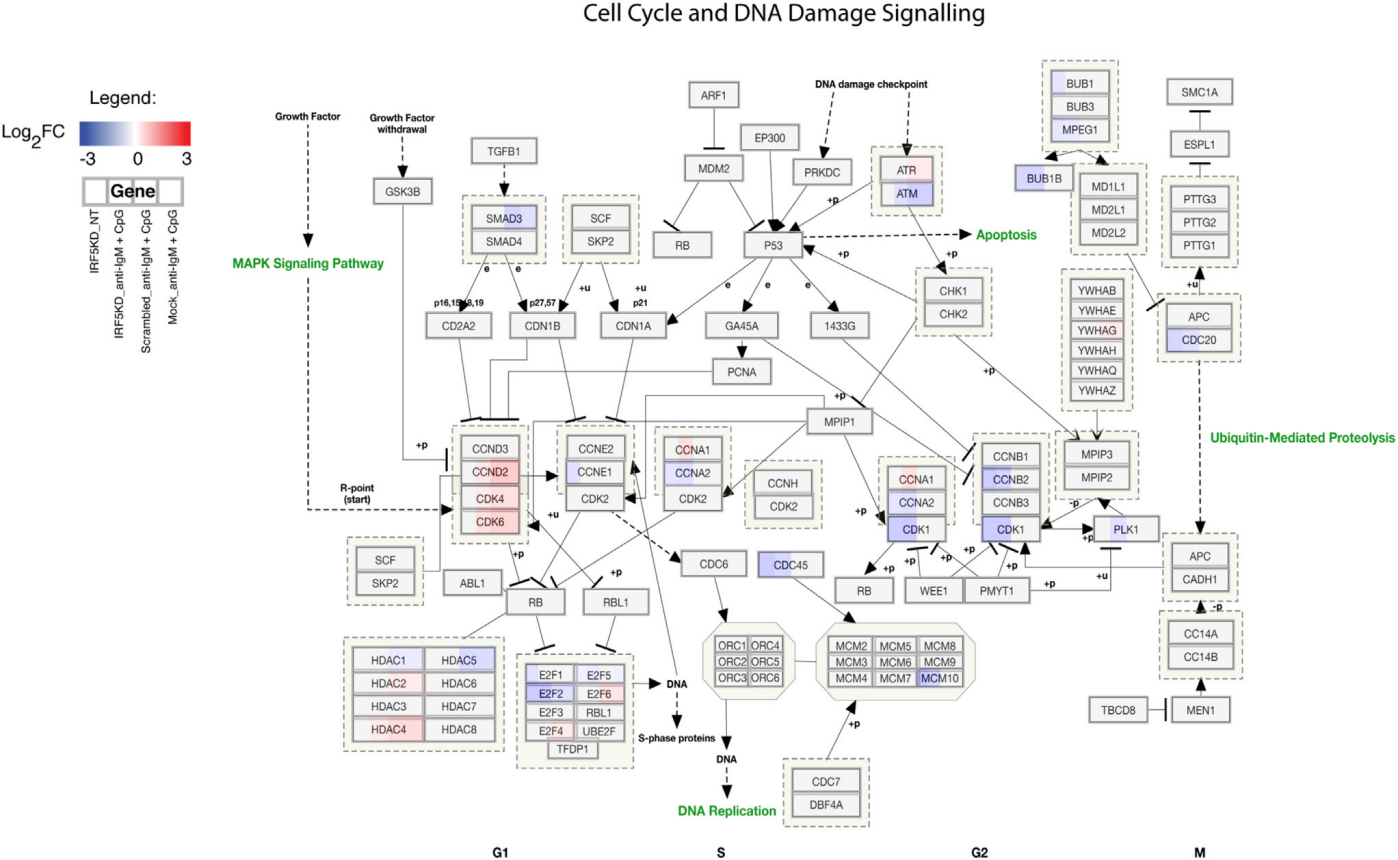
Interferon regulatory factor 5 regulates B cell proliferation and cell cycle. Similar to Figure 6C showing the overall downregulation of the cell cycle and DNA damage signaling pathway from ChIP-Seq and RNA-Seq datasets. Genes are shown as being either upregulated (red) or downregulated (blue) when compared to Mock untreated (NT). Representative expression is shown in four sections of each gene box as depicted in the Legend.

To gain a further understanding of IRF5-regulated pathways in primary naive B cells, we performed an in-depth integrative analysis of ChIP-Seq and RNA-Seq datasets. Results in Figures 6A,B are from pathway analysis showing the top 20 most activated pathways when compared to mock untreated samples. Data in Figure 6A show a distinct upregulation in the unfolded protein response, adipogenesis, and translation factor pathways after IRF5 knockdown. Conversely, knockdown of IRF5 in untreated and treated samples resulted in the downregulation of cell cycle and BCR signaling pathways (Figure 6B). Downregulation of these pathways coincide with functional defects detected after IRF5 knockdown (Figures 2 and 3). Given that the NF-κB signaling pathway has been shown to play a role in murine ASC differentiation (69), we mapped out genes in this pathway that were affected by IRF5 knockdown (Figure 6C). We also mapped out cell cycle and DNA damage signaling genes affected by IRF5 knockdown since we detected an alteration in BCR/TLR9-induced cell proliferation (Figure 7). Last, given that early defects in B cell activation were detected after IRF5 knockdown, we mapped out the complex interaction of genes involved in the BCR signaling pathway that were differentially affected (Figure S5 in Supplementary Material). Together, these data implicate IRF5 in the regulation of multiple signaling pathways involved in ASC differentiation and B cell survival.

## Discussion

The differentiation of ASCs is reliant upon a large network of transcription factors, which are responsive to various B cell activation pathways. Among the B cell activation pathways known to play a role in ASC differentiation is the TLR signaling pathway (6, 48). IRF5 is known to act downstream of TLR signaling in monocytes and dendritic cells, but characterization of IRF5 activation in human B cells has not been previously shown. Here we demonstrate that the transcription factor IRF5 acts downstream of TLR signaling to drive proliferation and differentiation to ASCs. However, unlike other transcription factors involved in ASC differentiation, such as *IRF4, PRDM1, XBP1*, and *MYC* that require early transcriptional upregulation (Figures 5E,F), IRF5 is uniquely poised for the rapid modulation of ASC-associated gene transcription in human B cells since its early regulation occurs through nuclear translocation (Figures 1A–C). Whether this effect is dependent on TLR9/BCR-induced B cell activation or would occur downstream of other B cell activating pathways is not currently known. However, since previous data in *Irf5^−^*^/^*^−^* mice showed a direct transcriptional role for IRF5 in the regulation of *PRDM1* and the γ*2a* locus (22, 25), it is intriguing to consider IRF5 as a global mediator of antibody responses. Our finding of significantly reduced plasmablast differentiation and IgG antibody production following IRF5 knockdown highlights the role of IRF5 in mediating antibody responses. Contrary to murine data, however, we were unable to detect IRF5 peak enrichment at *PRDM1* or any *IgG* locus by ChIP-Seq. This may be due to the early time point of analysis (4 h post-stimulation) or that these genes do not contain regulatory sequences recognized by IRF5. Similar to *Irf5^−^*^/^*^−^* mice, though, data presented in Figure 3G suggest a role for IRF5 in controlling IgG isotype secretion; however, IgG2 and 4 levels were secreted at significantly lower levels than IgG1 and 3 in response to stimulation. Additional work will be necessary to determine the role of IRF5 in human CSR.

The finding of reduced plasmablast differentiation in isolated naive B cells nucleofected with *IRF5* siRNA highlights a new B cell-intrinsic role for IRF5. These findings overlap with recent work in a murine model of lupus showing reduced ASCs in *Irf5^−/−^* MRL/*lpr* mice (20). However, it was not determined whether reduced ASCs was a result of IRF5 B cell-intrinsic or -extrinsic function. Data presented herein also point to IRF5 influences on late stage B cell effector functions. This is supported by the large difference in IgG isotype secretion as compared to intracellular IgG suggesting a potential role for IRF5 in secretion (Figures 3D–F).

Interferon regulatory factor 5 activity/function following nuclear translocation can occur through either transcriptional regulation or protein–protein interactions. We conclude from ChIP-Seq analysis that IRF5 regulates the transcriptional control of ASC differentiation. Through ChIP-Seq analysis, we found that IRF5 bound a wide range of target genes involved in proliferation, ASC differentiation, and cell cycle control. Among the most prominent targets were *ERK1, IRF4*, and *MYC*. Previous characterization of IRF5 transcriptional targets was in murine macrophages, revealing that IRF5 and NFĸB bound promoters of inflammatory cytokines (60). Earlier ChIP-Seq work in human PBMC found IRF5 to have overlapping transcriptional targets with STAT4 (70). Results presented herein represent the first characterization of IRF5 target genes in human primary B cells. In stark contrast to previous work, IRF5 regulated pathways in human B cells are focused on proliferation, survival, cell cycle, and ASC differentiation (Figures 6 and 7; Figure S5 in Supplementary Material).

Results from RNA-Seq further confirm an intrinsic role for IRF5 in ASC differentiation. Genes known to be critical in B cell proliferation and differentiation were drastically reduced following knockdown (Figure 5F). Our finding of decreased *PRDM1* expression was similar to previous work in *Irf5^−/−^* mice carrying mutant *DOCK2* (25, 26); however, we did not detect direct binding of IRF5 to the *PRDM1* promoter even though ISRE and IRF-E sites were identified (Table 3). Rather, data indicate that decreased *PRDM1* expression is a byproduct of other genes regulated by IRF5, such as *ERK1* and *MYC* (8, 62). It is intriguing to propose from these data that IRF5 may be a gatekeeper for antigen-stimulated naive B cells to enter the proliferation cycle. If true, then alterations in IRF5 activation during early stages of B cell activation may initiate the first break in self-tolerance. Current limitations in manipulating primary B cells, however, did not allow us to address whether the defect in B cell proliferation was due to reduced proliferation potential or reduced numbers of cells entering the cell cycle (Figures 3A,B). However, these data provide insight into other aspects of IRF5 function. For instance, the observed decrease in B cell proliferation suggests that the significant increase in IgD^+^ B cells detected after IRF5 knockdown is due to retention of IgD^+^ B cells rather than increased *de novo* synthesis since this would have been detected as an increase in proliferation rather than a decrease. Further, it implies that sufficient cell numbers were present for entering the cell cycle in response to BCR/TLR9 signaling. Instead, findings from pathway analysis of ChIP-Seq and RNA-Seq datasets support a key role for IRF5 in B cell proliferation *via* cell cycle regulation (Figure 7). Unfortunately, we were also unable to determine whether overexpression of MYC could restore ASC differentiation in IRF5 knockdown cells due to limitiations in transfection and/or viral infection efficiency (54, 55, 71). Complementary experiments in mice or CRISPR/Cas9 in primary naive B cells (72) will be required to further elucidate the mechanisms by which IRF5 controls early stages of ASC differentiation.

Last, results presented herein have direct implications for IRF5 function in SLE B cells. SLE patients carrying *IRF5* risk polymorphisms show elevated IRF5 expression in B lymphoblastoid cell lines (15, 16). SLE patients have elevated numbers of circulating ASCs that contribute to pathogenic autoantibody production and elevated circulating autoantibodies are present years before clinical symptoms (2, 4). It is intriguing to propose that polymorphisms in *IRF5* contribute to early skewing of B cell subset distribution and to breaks in self-tolerance which may ultimately lead to the observed increase in circulating ASCs and autoantibodies found in SLE patients.

## Ethics Statement

This study was carried out in accordance with the recommendations of the Rutgers Biomedical and Health Sciences IRB and the Feinstein Institute for Medical Research IRB with written informed consent from all subjects. All subjects gave written informed consent in accordance with the Declaration of Helsinki. The protocol was approved by the Rutgers Biomedical and Health Sciences IRB and the Feinstein Institute for Medical Research IRB.

## Author Contributions

SD and BB planned and designed the study. SD and TS performed the experiments. SD and BZ analyzed bioinformatics data. SD, SS, AW, BZ, RD, TS, and BB analyzed experimental data. SD and BB prepared the manuscript.

## Conflict of Interest Statement

BZ and AW were employed by Pfizer Inc. All other authors declare not competing interests.
