# Supplementary material for: B Cell-Intrinsic Role for IRF5 in TLR9/BCR-Induced Human B Cell Activation, Proliferation, and Plasmablast Differentiation
Source: Front Immunol. 2018 Jan 10;8:1938. doi: 10.3389/fimmu.2017.01938 (PMC5768180; doi:10.3389/fimmu.2017.01938)
Supplement: Supplementary file 1 [file Presentation_1.pdf]

Supplemental Data

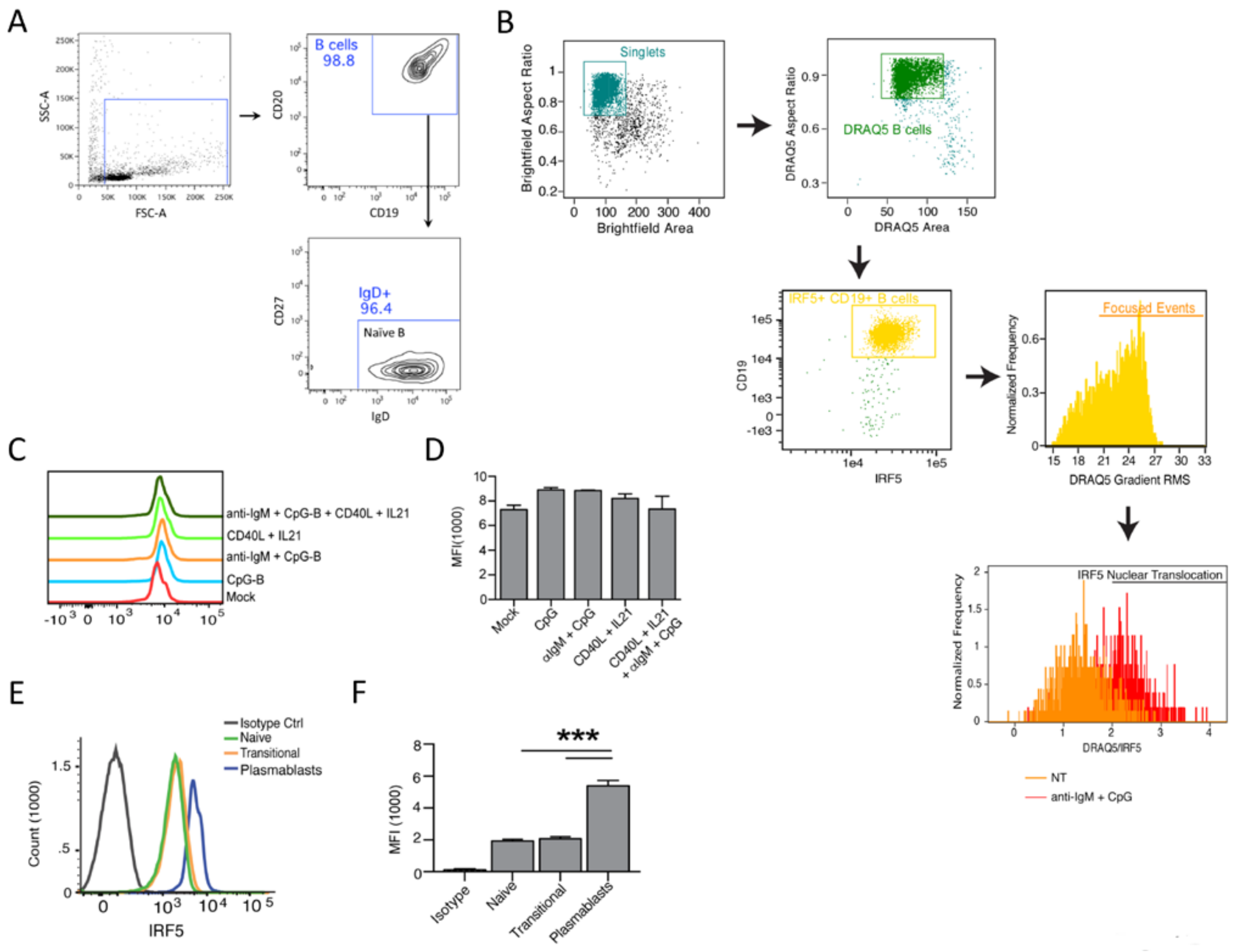

Figure S1. **IRF5 expression and activation in human primary B cells.** (A) Representative flow cytometry analysis of B cell purity after isolation gating on $CD19^{+}CD20^{+}C27^{-}IgD^{+}$ naïve B cells. (B) Quantification of IRF5 nuclear translocation through imaging flow cytometry. Representative gating scheme used to define IRF5 nuclear translocation in untreated (NT) and anti-IgM + CpG-B stimulated CD19+ B cells with an IRF5/DRAQ5 similarity score ≥2. (C) Representative flow cytometry histograms of IRF5 expression following stimulation with the indicated B cell activating stimuli for 2 h. Representative experiment from one donor is shown. (D) Same as in (C) except average MFI values of IRF5 expression following stimulation of B cells is shown from *n=3* independent donors. (E) Representative flow cytometry histogram of IRF5 expression in B cell subsets seen in peripheral blood of non-immunized healthy donors. (F) Average MFI of IRF5 expression in B cell subsets shown in (E). (Two-way ANOVA with Tukey's multiple comparison post-hoc test; *n=5* independent donors). Error bars represent standard deviation. ***$p \leq 0.001$.

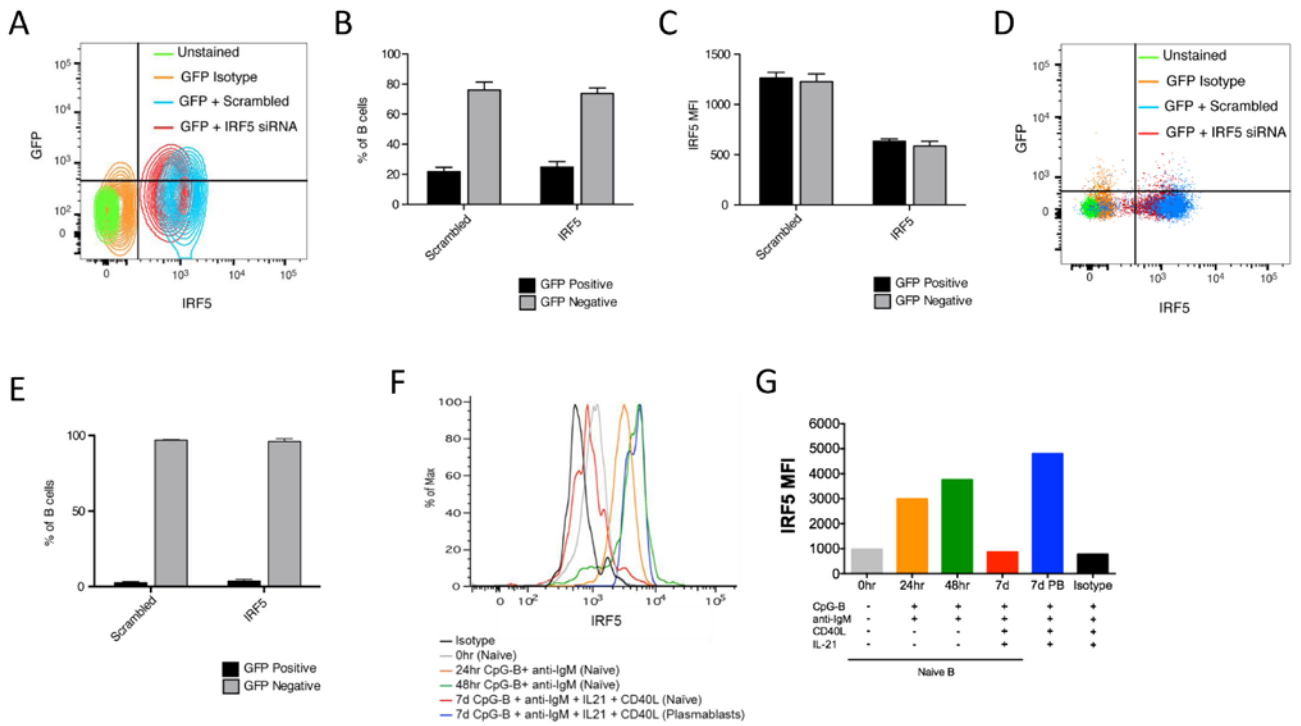

Figure S2. **Optimization of *IRF5* siRNA and GFP co-nucleofection in human primary naïve B cells.** (A) Representative contour plot from flow cytometry analysis of IRF5 expression and GFP expression in primary naïve B cells co-nucleofected with *IRF5* siRNA and GFP mRNA. (B) Percentage of B cells positive for GFP expression following nucleofection. (C) Average MFI values of IRF5 in GFP-positive or -negative B cells. (D) Similar to (A) except a representative dot plot from flow cytometry analysis of IRF5 expression and GFP expression from primary naïve B cells co-nucleofected with *IRF5* siRNA and pmaxGFP™ is shown. In this case, only dot plots were able to show GPF-positive cells. (E) Percentage of B cells positive for GFP expression in (D). Data show very low nucleofection efficiency of pmaxGFP™. (F) Representative flow cytometry histograms of IRF5 expression from *in vitro* cultured naïve B cells and resulting plasmablasts after 7 day culture. (G) Average MFI of IRF5 expression from (F). Error bars represent standard deviation from three independent replicates.

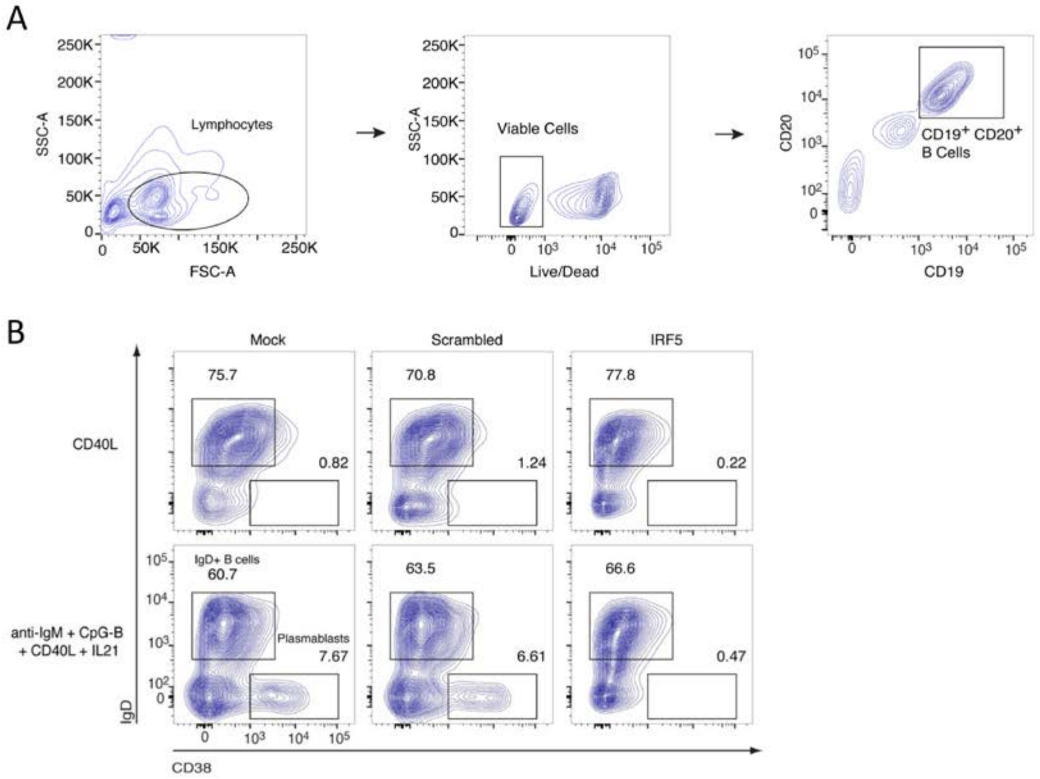

Figure S3. **Flow cytometry gating strategy to quantify plasmablast differentiation from *in vitro* culture.** (A) Representative gating strategy for viable B cells following knockdown of IRF5 and *in vitro* culture. (B) Alternative gating strategy for the identification of plasmablasts and IgD^+ naïve B cells.

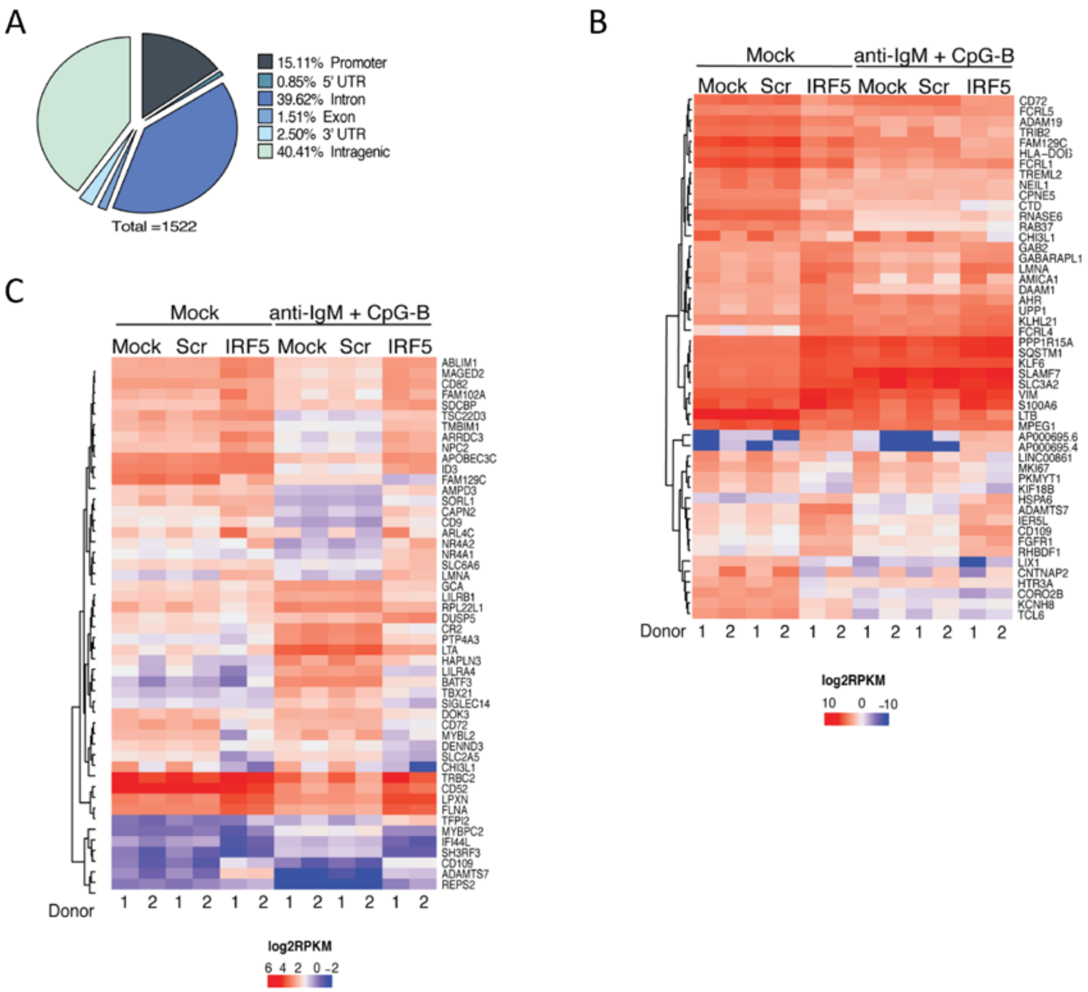

Figure S4. **IRF5 binding elements and significant gene expression changes associated with IRF5 knockdown and stimulation.** IRF5 ChIP was performed on the Ramos B lymphoblastoid cell line. Cells were either mock or anti-IgM and CpG-B stimulated for 4 h. Reads were mapped through BWA and peaks called through MACs. (A) Distribution of IRF5 mapped reads from ChIP-Seq of Ramos B cells. (B) Isolated naïve B cells were nucleofected with 500 nM of mock, scrambled or IRF5 siRNA and subsequently stimulated with anti-IgM and CpG for 6 h. RNA-seq was performed on B cells from n=2 independent donors. Heatmap of genes with greatest differential expression when normalized to untreated scrambled siRNA nucleofected control is shown. (C) Similar to (B) except heatmap of genes with greatest differential expression when normalized to anti-IgM and CpG-B treated scrambled siRNA nucleofected control is shown.

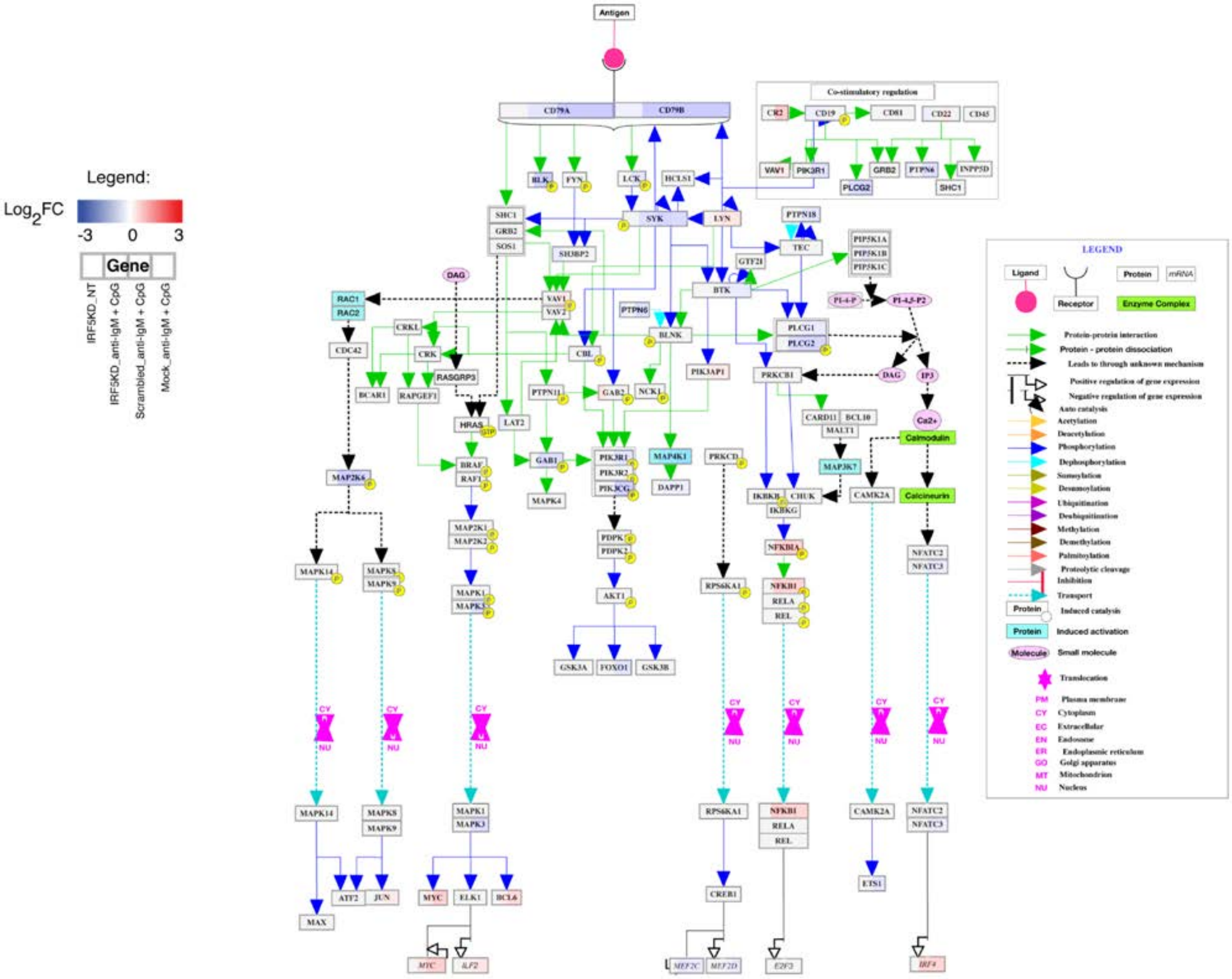

Figure S5. **IRF5 regulates the BCR signaling pathway.** Representative scheme showing the overall downregulation of the BCR signaling pathway after IRF5 knockdown. Boxes representing genes are color-coded based on the log2 fold-change values. Upregulated (red) or downregulated (blue) genes are shown as compared to Mock NT. Expression is shown by color code in four sections of each gene box. The gene box is divided into sections with one to one mapping comparisons of IRF5KD_NT, IRF5KD_anti-IgM+CpG, Scr_anti-IgM+CpG, and Mock_anti-IgM+CpG vs. reference Mock_NT, as shown in the upper left Legend. The Legend to the right of the figure defines the different mechanisms of pathway activation.
